# Tailoring Precursor‐Solvent Coordination Controls the Crystallization Kinetics and Nuclei Growth for Phase Homogenization in Wide‐Bandgap Perovskite Solar Cells

**DOI:** 10.1002/advs.202507660

**Published:** 2025-11-14

**Authors:** Saurabh Srivastava, Sudhir Ranjan, Harishankar Suman, Shailesh Kumar Sah, Shashikant Gupta, Subhakar Mangam, Shambhavi Rai, Jayant Jain, Srinivas Karthik Yadavalli, Shivam Tripathi, Anand Singh, Raju Kumar Gupta, Ashish Garg

**Affiliations:** ^1^ Department of Materials Science and Engineering Indian Institute of Technology Kanpur Kanpur 208016 India; ^2^ Department of Chemical Engineering Indian Institute of Technology Kanpur Kanpur 208016 India; ^3^ Department of Physics Indian Institute of Technology Roorkee Roorkee 247667 India; ^4^ Department of Sustainable Energy Engineering Indian Institute of Technology Kanpur Kanpur 208016 India; ^5^ Department of Materials Science and Engineering Indian Institute of Technology Delhi New Delhi 110016 India; ^6^ Department of Chemistry Indian Institute of Technology Kanpur Kanpur 208016 India; ^7^ Chandrakanta Kesavan Centre for Energy Policy and Climate Solutions Indian Institute of Technology Kanpur Kanpur 208016 India; ^8^ Centre for Environmental Science and Engineering Indian Institute of Technology Kanpur Kanpur 208016 India

**Keywords:** crystallization, halide homogenization, iodoplumbate, nucleation, phase transition, polytypes, wide bandgap

## Abstract

Wide‐bandgap (WBG) perovskites are promising top‐cell absorbers for tandem photovoltaics but suffer from unbalanced nucleation‐growth driven by strong coordination between polar aprotic solvents and perovskite precursors. This promotes the formation of hexagonal intermediate polytypes, stacking defects, halide‐cation migration, and phase segregation. Although long‐chain alkyl ammonium chlorides are used to control crystallization, the role of volatile ammonium chloride (AC) in altering the precursor chemistry and crystallization pathways in WBG perovskites remains unexplored. Present study, shows that AC weakens precursor‐solvent coordination and destabilizes undesired hexagonal polytypes. In situ characterizations indicate that AC induces high‐valence, de‐intercalated solvated iodoplumbate complexes that inhibit the sol–gel state and balance nucleation‐growth kinetics. Concurrently, Cl‐rich intermediates provide heterogeneous nucleation sites and, via cation exchange between NH_4_^+^ and Cs^+^/FA^+^ ions, retard uncontrolled crystal growth. The combined effect suppresses the formation of undesired phases, promotes transformation to the cubic perovskite phase, and enables defect self‐elimination during crystallization, yielding more homogeneous, higher‐quality films. As a result, AC‐treated perovskite films yield high‐quality 1.73 eV WBG perovskite solar cells with ≈18% PCE and a high *V_oc_* of 1.22 V, along with enhanced photostability.

## Graphical abstract

Volatile ammonium chloride (AC) regulates precursor‐solvent coordination and destabilizes hexagonal polytypes, promoting cubic‐phase formation and in situ defect self‐elimination in 1.73 eV wide‐bandgap perovskites. AC induces a de‐intercalated high‐valence iodoplumbate complex that suppresses the sol–gel state and balances nucleation, while Cl‐rich intermediates and $NH_{4}^{+}-Cs^{+}/FA^{+}$ cation exchange slow crystal growth, yielding devices with improved performance and excellent photostability.

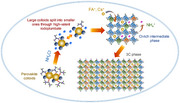

## 1 Introduction

Single junction perovskite solar cells (PSCs) have seen tremendous enhancement in the power conversion efficiencies (PCE) from ≈3.8% to 26.7% just within a decade, leveraging excellent optoelectronic properties of perovskite materials such as high absorption coefficients, long charge‐carrier diffusion lengths, simple solution processing, low levels of electronic disorder, etc.^[^ ^1^, ^2^, ^3^, ^4^ ^]^ Research has demonstrated that compositional manipulation of I and Br at the X site results in tuning of the optical bandgap (E_g_) of the mixed‐halide perovskites between 1.6 and 2.3 eV, thereby making them an excellent candidate for developing multi‐junction or tandem solar cells.^[^ ^5^, ^6^, ^7^, ^8^ ^]^ In recent years, wide‐bandgap (WBG) mixed‐halide perovskites with *E_g_* ≈1.7–1.8 eV have gained attention for their application as an absorber layer in the top cell of tandem solar cells with narrow‐bandgap silicon or CIGS or perovskite solar cells constituting the bottom part, enabling these solar cells to overcome the Shockley–Queisser limit of ≈30% PCE for single junctions.^[^ ^8^, ^9^, ^10^, ^11^ ^]^

However, mixed halide‐cation WBG perovskite absorbers exhibit shorter diffusion lengths and a significant open‐circuit voltage (*V_oc_*) deficit of ≈0.5 V, which is ≈0.3–0.4 V higher than in conventional single junction PSCs incorporating perovskites having a bandgap < 1.65 eV.^[^ ^12^, ^13^, ^14^ ^]^ This *V_oc_* deficit is attributed to the non‐radiative losses induced by heterogeneous crystallization, which leads to lattice strain, randomly oriented grains, and grain boundary defect terminations. These defects promote ion migration and accelerate material degradation,^[^ ^15^, ^16^, ^17^ ^]^ while also introducing photoinduced halide segregation, wherein mixed iodide‐bromide phases reversibly segregate into Br‐rich and narrow bandgap I‐rich domains that act as non‐radiative recombination centers, thereby limiting *V_oc_* and reducing device lifetimes.^[^ ^14^, ^18^ ^]^ In addition, rapid crystallization of Br‐rich phase exhibits a non‐homogeneous distribution of phases and compositional gradients across the film.^[^ ^19^, ^20^ ^]^ Since, phase homogenization often accompanies the crystallization process during film formation for achieving target stoichiometry, crystal growth gets interrupted, further creating halide migration channels. Therefore, there is a need to concentrate on the precise control of crystallization kinetics to obtain high‐quality WBG perovskite films.

During film formation, the kinetics of nucleation and subsequent growth of crystallites strongly affect the film's structure and its properties. During phase formation and its evolution, halide‐cation WBG perovskite precursor solution preferentially nucleates as hexagonal 2H phase, following a specific crystallization pathway of 2H‐4H‐6H‐3R(3C) (C: cubic, H: hexagonal, R: rhombohedral).^[^ ^21^ ^]^ However, the conversion of precursor to crystal involves complex phase‐conversion pathways, during which dynamic nucleation/growth processes lead to spontaneous generation of in situ stacking defects and dislocations. These defects serve as recombination centers for the charge carriers, ultimately limiting the device performance.^[^ ^22^ ^]^ Compared to MA (methylammonium) and FA (formamidinium) based perovskites, compositional complexities in WBG perovskite precursor ink formulation make the crystallization process uncontrolled, leading to the growth of compositionally distinct phases.^[^ ^23^ ^]^ Attempts have been made to regulate the formation dynamics of the 2H phase in FAPbI_3_ by introducing alkyl ammonium salts. These strategies facilitated the direct nucleation of 3C FAPbI_3_ while simultaneously lowering the phase transition temperature, resulting in enhanced crystallinity and a preferred crystal orientation in the films.^[^ ^24^ ^]^ However, using long‐chain volatile alkyl ammonium salts (MACl; methyl ammonium chloride and FACl; formamidinium chloride) as additives in WBG perovskite resulted in the formation of chloride‐rich hexagonal intermediate polytypes and 3C phase in the final films.^[^ ^25^, ^26^ ^]^ Recently, Chen et al. demonstrated a nuclei engineering approach to promote nucleation of halide homogenized 3C phases in prevalence to Br‐rich and 2H phase nuclei for compositionally independent WBG perovskite absorbers.^[^ ^27^ ^]^ Although high‐efficiency perovskite films have been obtained through these approaches, these studies have mainly relied on using N, N‐dimethylformamide (DMF) and dimethyl sulphoxide (DMSO) as primary polar aprotic solvents, which often results in poor photostability.

Meanwhile, strong coordination between perovskite precursor and DMF:DMSO, predominantly forms PbI_2_‐DMSO adduct (D‐complex) that extends the lifetime of sol–gel state, further aggravating the formation of multiple compositionally distinct hexagonal polytypes and 3C phase nuclei during the crystallization process.^[^ ^28^, ^29^, ^30^ ^]^ Elimination of these intermediate phases requires weakening solvent‐perovskite precursor coordination either by utilizing strongly coordinating additives (with Pb^2+^) with the ability to form a stable intermediate phase, or by replacing DMF/DMSO with solvents exhibiting weaker interaction with perovskite precursors. Building on this, several studies have demonstrated direct crystallization of MAPbI_3_ and FAPbI_3_ without the formation of intermediate polytype complexes.^[^ ^31^, ^32^ ^]^ The solvent evaporation rate has also been found to dictate nucleation‐growth kinetics and phase transition pathways of perovskites. This has often led to dense perovskite films but with poor crystallinity. To date, only one such study has been reported in WBG perovskites, where Guaita et al. used N‐methyl‐2‐pyrrolidone (NMP) as a solvent with MACl as an additive to eliminate the intermediate phase in gas‐quenched films, promoting direct crystallization into the perovskite 3C phase.^[^ ^33^ ^]^ This destabilization of the intermediate phases facilitated halide homogenization, improving photostability and device performance.

Despite significant advancements, the role of the surrounding chemical environment and precursor‐coordinating molecular interactions in WBG perovskite precursor solutions remains unexplored. A deeper microscopic understanding of the nucleation and growth kinetics during phase transformation and their effect on defect formation in WBG perovskite is still lacking. Coordinated iodoplumbate complexes with DMF/DMSO often lower the crystallization energy for film formation, but their coordination number is extremely sensitive to solution chemistry and storage conditions, often resulting in misoriented films.^[^ ^34^, ^35^ ^]^ Therefore, this warrants precise control of environment‐sensitive iodoplumbate(solvent)_x_ complex to establish a deeper understanding of crystallization pathways to better regulate nucleation and growth kinetics so that one can form high‐quality perovskite films with lesser intrinsic defects.

It is with the above objective, in this work, that we have systematically investigated the critical role of volatile ammonium chloride (AC) as an additive on the crystallization dynamics, phase transformation pathways, and their effect on the key properties of the FA_0.8_Cs_0.2_Pb(I_0.7_Br_0.3_) WBG perovskite and the photovoltaic devices thereof. We have used time‐resolved temperature‐dependent in situ photoluminescence (PL) and grazing‐incidence X‐ray diffraction characterization for studying the nucleation and phase evolution during the spin‐coating and annealing stages of the film. Our results demonstrate that incorporating AC as an additive weakens the interaction between uncoordinated lead and solvent, leading to the formation of a stable high‐valence de‐intercalated solvated iodoplumbate complex. This modification in the perovskite precursor interaction promotes balanced crystallization kinetics, suppressing the formation of intermediate hexagonal phases, thereby driving the direct formation of pure perovskite 3C phase with enhanced crystallinity. Through detailed characterization, this study sheds light on how carefully tailoring perovskite precursor‐solvent coordination chemistry via the introduction of volatile ammonium chloride enables precise control over crystallization kinetics in WBG perovskites. This approach facilitates the self‐elimination of stacking defects and mitigates microstrain during crystallization, resulting in suppressed phase segregation, improved morphology, and reduced defect density. The strategy of in situ self‐elimination of stacking defects using AC‐based additives enabled WBG PSCs to achieve a maximum PCE of ≈18% with improved photostability. Therefore, this study proposes a strategy for the rational design of crystallization pathways for in situ suppression of stacking defects for improving the performance and photostability of WBG PSCs.

## 2 Results and Discussion

### 2.1 Understanding Perovskite Precursor‐Solvent Coordination Chemistry

During thin film processing, balancing nucleation and diffusion‐controlled growth kinetics is crucial for achieving high‐quality films. Understanding and controlling this process is essential, particularly in the processing of perovskite thin films, as it involves complex phase transformation pathways influenced by the choice of solvent, additives, and dopants, etc.^[^ ^36^, ^37^ ^]^ For example, crystallization kinetics are strongly controlled by the solvent's boiling point and its interaction with perovskite precursors. Most perovskite precursors are prepared using DMF/DMSO, as these solvents exhibit strong coordination with Pb^2+^ ions. However, this strong coordination hinders the nucleation of perovskite crystals, resulting in incomplete coverage and macroscopic film heterogeneities.^[^ ^38^ ^]^ Furthermore, owing to the high boiling point of DMSO and its stronger coordination with Pb^2+^ ions, it gets trapped in the annealed perovskite film, especially at the buried interface, acting as a deep trap for carrier recombination. Although this problem can be overcome by reducing DMSO content, it often compromises the crystallization process of the perovskite film, ultimately degrading the device performance.^[^ ^39^ ^]^

**Figure 1:**
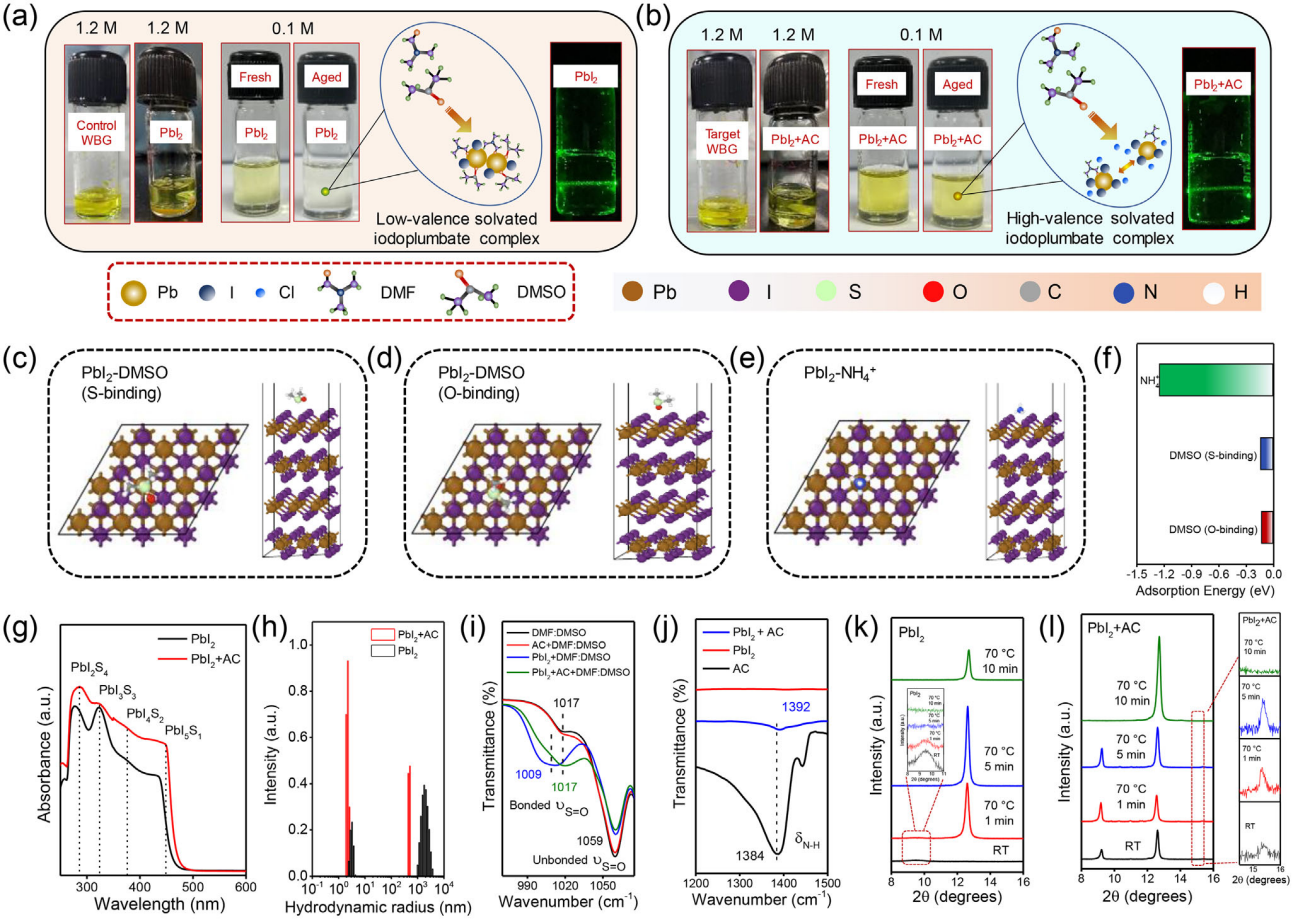
a) Photographs of the vials containing 1.2 m WBG control perovskite precursor solution (turbid), 1.2 m PbI_2_ solution (slow dissolution), 0.1 m PbI_2_ solution in DMF:DMSO (4:1), and respective photograph of the vial showing laser scattering from low‐valence solvated iodoplumbate complexes present in 0.1 m PbI_2_ solution. b) Photographs of the vials containing 1.2 m WBG target perovskite precursor solution (clear), 1.2 m PbI_2_+AC solution (fast dissolution), 0.1 m PbI_2_+AC solution in DMF:DMSO (4:1), and respective photograph of vial showing laser scattering from high‐valence solvated iodoplumbate complexes present in 0.1 M PbI_2_+AC solution. Schematic representations of the possible interaction configurations of PbI_2_ with c) DMSO via S‐binding, d) DMSO via O‐binding, and e) ${\mathrm{NH}}_{4}^{+}$, as obtained from DFT modelling. f) Corresponding adsorption energy values for the three configurations, presented as a bar graph. g) Absorbance spectra of diluted (0.1M) PbI_2_ and PbI_2_+AC solutions highlighting the presence of different iodoplumbate complexes. h) Dynamic light scattering results showing the formation of larger colloids in PbI_2_ and smaller colloids in PbI_2_ with AC solutions, respectively. i) Fourier transform infrared (FTIR) spectra of DMSO, PbI_2_ in DMSO, AC in DMSO, and PbI_2_ with AC in DMSO showing interaction of AC with PbI_2_ via the shifting of the bonded S═O stretching peak. j) FTIR spectra of AC, PbI_2_ and PbI_2_ with AC showing the shifting of N─H bending peak. k,l) XRD patterns of the PbI_2_ and PbI_2_+AC films annealed at 70 °C for different time intervals. The peak positioned at 15.4° in PbI_2_+AC films belonging to NH_4_Pb(I_x_Cl_1‐x_)_3_ is shown in the zoomed‐in XRD pattern.

In this work, we discovered that incorporating AC as an additive effectively tailored the coordination chemistry between the WBG perovskite precursor and solvent, thereby regulating crystallization and solvent volatilization. The chemical structure of AC is shown in Figure S1 (Supporting Information). To gain insight into the mechanism of AC's role in regulating the perovskite crystallization, we first analysed the interaction between the PbI_2_ precursor and AC by preparing 0.1 and 1.2 m PbI_2_/DMF:DMSO solutions with and without AC. First, 1.2 m solutions were stirred and heated to 70 °C for 3 min. After cooling to room temperature (**Figure** **1**a,b), PbI_2_‐AC solution demonstrated enhanced solubility of solutes, as evident from a clear, yellow‐colored liquid solution with a minimal amount of solute left at the bottom. However, a substantial amount of residual solute precipitated out in the pure PbI_2_ solution. A similar trend of increased dissolution was observed for the perovskite precursor solution with AC addition.

Next, we found that 0.1 m PbI_2_ dissolved quickly in solution with AC at room temperature when shaken for 30 s, whereas pure PbI_2_ resulted in a turbid light‐yellow solution. Interestingly, after resting for 5 min, the pure PbI_2_ solution turned transparent, while the PbI_2_‐AC solution retained its original clear yellow color. These findings suggest strong coordination between PbI_2_ and AC, which leads to the formation of an intermediate phase of x[NH_4_^+^][PbI_2_Cl_x_]^x−^, thereby enhancing the solubility of PbI_2_ in DMF:DMSO solvent.^[^ ^40^ ^]^ Previous studies have shown that perovskite precursors exist as colloidal dispersions in the mother solution, where these colloids could be considered as frameworks of lead polyhalides. The coordination degree of these colloids significantly impacts the film properties. The colloidal nature of the solutions was confirmed through a Tyndal effect experiment, which demonstrated light scattering in a 0.1 m solution. PbI_2_‐AC solution exhibited relatively weaker and ordered light scattering, indicating the presence of smaller Pb‐I clusters (colloids) suspended in the solution. The smaller Pb‐I cluster size is attributed to chlorine‐induced nucleation and the formation of an intermediate phase, facilitated by the bond formation between NH_3_····H^+^─Cl^−^ and I‐Pb‐I. This intermediate phase is expected to weaken the coordination between PbI_2_ and DMF:DMSO, thereby promoting the rapid dissolution of PbI_2_.^[^ ^41^ ^]^ The intermediate phase formation was identified by XRD analysis, discussed in a later section. The degree of complexation of solvated iodoplumbate complexes in the perovskite precursors is of great relevance in determining the final optoelectronic properties of the perovskite thin films.^[^ ^42^ ^]^ Density functional theory (DFT) calculations were performed to investigate the effect of AC addition on the adsorption energy of PbI_2_ with DMSO. The theoretical simulations indicate that DMSO can adsorb on PbI_2_ (0001) through either the O or S atom, with the S‐site configuration having slightly higher adsorption energy, making S‐site binding more stable than the O‐site binding. In contrast, AC exhibits much stronger adsorption on PbI_2_, with an adsorption energy nearly ten‐fold higher (‐1.23 vs ‐0.11 eV) as compared to DMSO adsorption on PbI_2_. The stable adsorption configurations and corresponding energies of DMSO and AC on the hexagonal PbI_2_ (0001) surface obtained from our DFT calculations are shown in Figure 1c‐f. The stronger binding of PbI_2_ with AC relative to DMSO as revealed from DFT calculations is consistent with the experimental results. The optical absorption spectra of 0.1 m solutions show that introducing AC caused a redshift in the absorption maximum wavelength of PbI_2,_ with a sharp colloidal absorption edge (Figure 1g). This redshift indicates a stronger interaction between AC and undercoordinated lead while weakening Pb‐solvent coordination. Reduced solvent coordination allows more I^−^ ions from other iodoplumbate molecules to interact with the central Pb^2+^ ions, leading to the formation of stable high‐valent iodoplumbate complexes.^[^ ^43^ ^]^ This finding suggests that AC stabilizes the colloidal system, corroborating the previously discussed observation that the aged PbI_2_‐AC solution retains its original color.

Previous studies suggest that the presence of high‐valent iodoplumbates lowers the activation energy for transformation from perovskite precursor phase to 3C perovskite, potentially yielding films with higher crystallinity and improved morphology. To further investigate the effect of AC on colloidal behavior, we conducted dynamic light scattering (DLS) measurements to analyze the particle size distribution in PbI_2_ solutions. Compared to pure PbI_2_ precursor, PbI_2_‐AC solution exhibited smaller colloids with much narrower distribution, suggesting the presence of high‐valent iodoplumbates (Figure 1h). The uniform small‐sized colloids containing high‐valent iodoplumbates in PbI_2_‐AC solutions serve as templates for the growth of highly crystalline perovskite, enabling balanced nucleation‐growth kinetics, as confirmed by SEM images and XRD results.

To further confirm the reduced interaction between PbI_2_ and DMF:DMSO in the presence of AC, Fourier transform infrared (FTIR) spectroscopy was performed. The FTIR spectra (Figure 1e) show two peaks for DMSO at 1059 and 1017 cm^−1^, respectively, corresponding to the vibration stretching of free and bonded S═O bonds, respectively.^[^ ^44^ ^]^ The shoulder peak at 1017 cm^−1^ gradually shifted to 1009 cm^−1^ for pure PbI_2_ in DMF:DMSO solution, attributed to an increase in the electron cloud density near the oxygen atom after interaction with the less electronegative lead atom. However, in the PbI_2_‐AC solution, the peak position remained unchanged, indicating the weak coordination ability of PbI_2_ with DMSO in the presence of AC. A similar trend (Figure S2a, Supporting Information) is observed for the C═O stretching vibration peak of DMF, further confirming that AC weakens the coordination between PbI_2_ and solvent molecules. This inhibits the formation of [PbI_2_(solvent)_x_] adduct, leading to an increase in the number of free solvent molecules.

Further, the influence of AC on the coordination ability of DMF:DMSO with FAI is investigated. As observed from FTIR spectra in Figure S2b (Supporting Information), no significant shift is observed in the bonded S═O vibration peak for solutions containing both FAI and AC, indicating a very weak interaction of these species with DMF:DMSO. However, the addition of AC to FAI in DMSO did not change the position of the bonded S═O vibration peak compared with that of DMSO, suggesting AC does not affect the coordination chemistry between FAI and solvent molecules. This observation is consistent with the photograph in Figure S2c (Supporting Information), where a residual white solid is obtained in the solution containing a mixture of AC and FAI. ^1^H NMR analysis was conducted to examine the interaction between FAI and AC. As observed from the ^1^H NMR spectra shown in Figure S3 (Supporting Information) for the mixed sample, both FA^+^ (δ = 8.78 and ∼7.9 ppm) and NH_4_^+^ (δ ≈ 7.2 ppm) retained their characteristic resonance peak position without the appearance of any additional signals, indicating no significant chemical reaction between FAI and AC. However, a feeble upfield shift in the FA^+^ resonance signal (δ = 8.78 to δ = 8.95 ppm) was observed, attributed to very weak ionic interactions or hydrogen bonding, suggesting the presence of residual solid in the FAI+AC solution. Additionally, FTIR spectra in Figure S2d (Supporting Information) exhibit a greater shift in C═N vibration peak for the mixture of AC and FAI in DMF:DMSO (1721 cm^−1^) compared to FAI alone (1725 cm^−1^), confirming an interaction between AC and FAI to some extent, which is in agreement with NMR results. A similar trend is observed for the solution containing a mixture of PbBr_2_ and AC, as shown in Figure S4 (Supporting Information). Moreover, FTIR spectra of the pristine PbI_2_ and the PbI_2_‐AC film exhibit a redshift in the N─H stretching peak (Figure 1j), suggesting hydrogen bond formation between the NH group and I^−^ in PbI_2_, corroborating the strong coordination between AC and PbI_2_.

To investigate the intermediate phase and verify the reduction in coordination effect between PbI_2_ and DMF:DMSO due to AC, we performed X‐ray diffraction (XRD) analysis on as‐prepared PbI_2_ solid‐state films. Figure 1k presents the time‐dependent evolution of XRD spectra for PbI_2_ films annealed at 70 °C to remove volatile DMF and partially evaporate DMSO. The XRD analysis shows that the D‐complex (corresponding to diffraction peak at ≈9.4°) gradually transforms to crystalline PbI_2_ (peak at ≈12.6°) and gets eliminated after 1 min of annealing at 70 °C. Interestingly, no D‐complex phase is observed in the PbI_2_‐AC sample (Figure 1l). Instead, intermediate phase peaks at ≈9.2° and 15.4°, corresponding to x[NH_4_^+^][PbI_2_Cl_x_]^x−^, are detected at room temperature, along with a strong PbI_2_.^[^ ^45^ ^]^ The intermediate phase slows down the crystallization of perovskite crystals, leading to the formation of high‐quality films. The intermediate phase continuously grew with time but was undetected after 10 min of annealing, most likely due to the release of NH_3_ and HI (HCl) as the byproducts. Additionally, with time, PbI_2_‐AC shows a shift toward a higher Bragg's angle corresponding to the (001) plane of PbI_2_ (Table S1, Supporting Information), suggesting enhanced de‐intercalation of DMSO from the D‐complex, resulting in a smaller d‐spacing. The absence of the D‐complex phase in the PbI_2_‐AC sample suggests that AC facilitates the rapid formation of crystalline PbI_2_, indicating AC effectively removes DMSO from the film by weakening its interaction with PbI_2_. Additionally, the smaller d‐spacing in the PbI_2_‐AC sample also clearly implies the ability of AC in removing DMSO from the film by weakening the interaction of PbI_2_ with DMSO. From all these results, we conclude that AC interacts with the Pb^2+^ ions in the perovskite precursor while simultaneously competing with DMF:DMSO in the solution.

### 2.2 Structural Evolution During Film Formation

**Figure 2:**
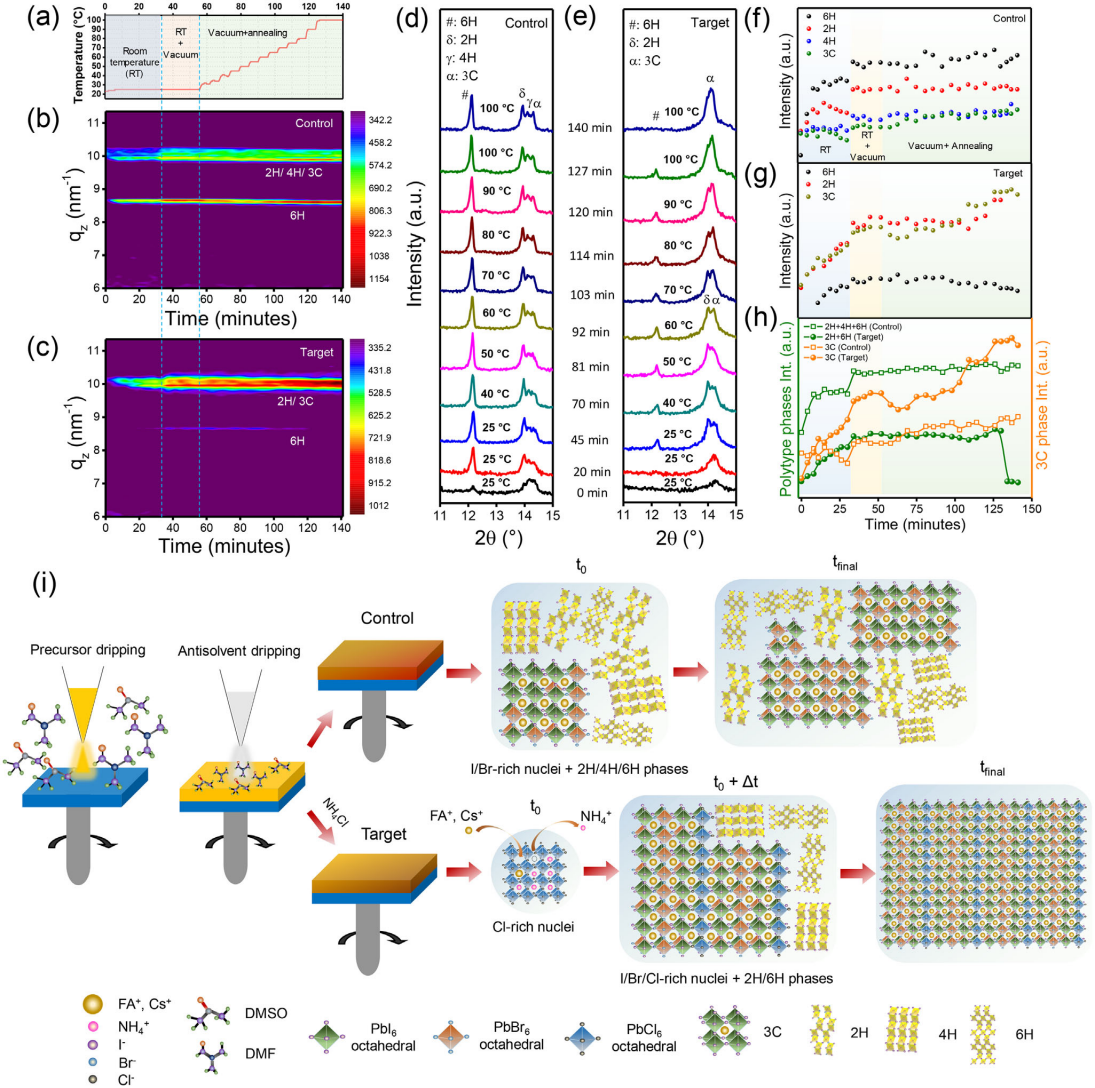
a) Temperature versus time profile of the in situ X‐ray diffraction measurements indicating the three steps in measurement: XRD scans at 25 °C, XRD scans at 25 °C under vacuum, and XRD scans under vacuum‐annealing. In situ XRD peak intensity maps of the b) control and c) target perovskite films showing the formation of 2H/4H/6H and 3C perovskite phases. d,e) Time‐evolution of the 2H/4H/6H and 3C phases in the control and target perovskite films shown at different temperatures (25°–100 °C). XRD peak intensity at different time intervals shows the changes in 2H/4H/6H and 3C phases in the f) control and g) target perovskite films. h) Time‐dependent change in the XRD peak intensity ratios of 2H/4H/6H and 3C phases of the control and target perovskite films. i) Schematic representation of crystallization modulation in WBG perovskite films.

To understand the influence of the AC additive on phase evolution of the perovskite films, we performed in situ grazing incidence XRD (GIXRD) experiments, tracking changes over time and temperature for films with (target) and without (control) the AC additive. As mentioned in the experimental section, the in situ GIXRD measurements were performed in three stages: (I) at room temperature, (II) at room temperature under vacuum conditions; and (III) under vacuum annealing to replicate the actual film formation process, as shown in **Figure** **2**a, illustrating the temperature versus time profile.

Figure 2b,c illustrates the integrated intensity 2D maps of the GIXRD patterns, converted from the in situ temperature‐ and time‐dependent GIXRD analyses. These figures clearly show that the target sample has a very feeble peak of the 6H phase, while a stronger and narrower peak for the 2H/4H/3C phase in comparison to the control sample. The time evolution of the diffraction patterns for the control and target perovskite films at different temperatures is shown in Figure S5 (Supporting Information). The XRD pattern shown in Figure 2d shows that the as‐cast spin‐coated control film immediately crystallized into a Br‐rich 3C perovskite phase. Additionally, several photoinactive polytype intermediates formed with reflections corresponding to the 2H, 4H, and 6H polytype phases at room temperature. It should be noted that the 4H and 6H polytypes are associated with the mixed‐halide perovskites (Br‐I), while 2H corresponds to pure iodide‐based perovskites. In the control film, the 3C perovskite phase peak is less intense and has a broader FWHM compared to the target film (discussed later in the section). This weaker crystallinity is likely due to the presence of intermediate phases that compete with the 3C perovskite phase, hindering the growth of the perovskite nanocrystalline structure.

Meanwhile, with the addition of AC, a sharp 3C phase peak located at a higher Bragg angle compared to that in the control film is observed in the as‐cast spin‐coated target film without any formation of polytype intermediates (Figure 2e). The shift in the Bragg angle toward a higher value suggests a smaller unit cell, indicating that the 3C phase in the target film is likely the Cl‐rich perovskite phase. The direct crystallization of this Cl‐rich phase, without the formation of intermediate phases, can be attributed to the stronger Pb─Cl bonds compared to the weaker Pb‐DMSO and Pb‐I/Br bonds.^[^ ^21^ ^]^ The necessity for out‐diffusion of Cl^−^, whether as a prerequisite for nucleation or a byproduct of growth, creates a kinetic barrier that prevents the crystallization of intermediate polytype phases and promotes the orderly growth of the perovskite lattice.^[^ ^23^ ^]^

These findings clearly indicate that AC facilitates the initial nucleation of Cl‐rich perovskite and suppresses the intermediate‐phase formation. In stage II, when vacuum is applied at room temperature, the peak position of the Br‐rich 3C perovskite phase in the control film shifts to a lower diffraction angle owing to the diffusion of I^−^ into the initially formed Br‐rich nuclei phase. However, in the target film, competing hexagonal polytype phases 2H and 6H emerged, while the 4H phase was suppressed. Additionally, the Cl‐rich perovskite 3C phase peak shifts to a lower Bragg angle. The emergence of 2H/6H phases indicates further nucleation of Br and I‐rich perovskites, while the shift toward the lower Bragg angle indicates diffusion of Br/I in the initially crystallized cubic perovskite phase. Meanwhile, 2H/6H phase peaks appeared at a higher Bragg angle than in control films, signifying the formation of the Cl‐incorporated 2H/6H phases. As time progresses through Stage II and III, the 3C phase peaks in both films continue to shift to lower Bragg angles, which can be attributed to the migration of I^−^ ions into the Br‐rich nuclei before halide homogenization occurs in the target film. Figure 2f,g show how the diffraction peak intensities of the 3C perovskite and photoinactive intermediate hexagonal polytype phases evolve over time in both control and target films. The changes in these peaks during the three stages of film formation highlight distinct crystallization behaviors between the two films. As shown in Figure 2h, the weight fractions of 3C and polytype phases increased continuously in the control films. However, polytype phases remained dominant throughout the experiment, indicating the crystallization of competing crystalline phases. In general, the polytype phases, particularly the 6H phase, form more readily than the 3C phase, indicating that the hexagonal phases are more stable.

To understand the reason for the predominant presence of intermediate phases, we performed ex situ GIXRD during spin coating. Figure S6 (Supporting Information) illustrates that during the first 20 s of spin coating, the diffraction pattern is dominated by a sharp peak resembling a semicrystalline sol–gel state. This sol–gel state indicates the presence of a solvated non‐perovskite precursor phase, resulting from strong coordination between solvent (DMF/DMSO) and the perovskite precursor. The strong interaction between the perovskite precursor and the solvent prolongs the sol–gel state, preventing its collapse and promoting the growth of multiple compositionally distinct phases.^[^ ^23^ ^]^ In target films, the chloride‐rich 3C‐phase crystallized immediately, followed by the formation of polytypes (2H and 6H), while the 4H polytype was suppressed during stage I. The fractions of 3C‐ and polytype phases increased sharply until the end of stage II, indicating competitive growth of crystalline phases, consequently leading to the preferential crystallization of the 2H phases over 6H and 3C phases. During stage III of vacuum annealing, the 3C phase continued to grow while the 2H phase reduced, suggesting that the 3C phase formed at the expense of polytype phases. The inhibited growth of polytype phases can be attributed to the weakened perovskite precursor‐solvent interaction, which suppresses gel state formation as observed in Figure S6 (Supporting Information). This suppression leads to the rapid intercalation of cations into the inorganic framework, regulating the crystal growth rate and resulting in perovskite films with desirable phase, texture, and microstructure.^[^ ^46^, ^47^, ^48^, ^49^ ^]^

Notably, the target films exhibited a faster disappearance of hexagonal polytypes from the initial nuclei, suggesting a reduced barrier for I/Br migration into the initially formed 3C perovskite phase. This migration replaces Cl in the growing cubic perovskite phase, leading to a well‐crystallized 3C phase with a uniform halide distribution. These findings demonstrate that the addition of AC tailors the perovskite precursor‐solvent interaction, suppressing the formation of hexagonal intermediate phases and balancing overall crystallization kinetics. This ultimately promotes the nucleation of the crystalline 3C phase as depicted in the schematic shown in Figure 2i. Further, thermogravimetric analysis (TGA) also confirms that precursor‐solvent coordination is weakened in the presence of AC, as shown in Figure S7 (Supporting Information). The target precursor exhibited a pronounced and more rapid DMSO loss at ≈169 °C, compared with ≈182 °C for the control. This ≈13 °C downward shift in the DMSO volatilization temperature signifies a reduced DMSO‐PbI_2_ binding strength, i.e., a lower energy barrier for solvent release.

### 2.3 Understanding the Nucleation and Growth Mechanism

#### 2.3.1 In Situ Photoluminescence Study

We performed in situ photoluminescence (PL) studies to systematically investigate the role of AC on the nucleation and crystallization of WBG perovskites in real time. In situ PL measurement is a widely used spectroscopy technique for tracking bandgap variations due to halide ratios in halide perovskites, as well as the evolution of crystalline phase and nanocrystal sizes during growth. Our custom‐built setup, comprising a diode laser source (λ_ex_ = 450 nm, 8 mW cm^−2^) integrated with a spectrophotometer, housed inside a nitrogen‐filled glovebox, was used to track PL evolution during crystallization processes. The PL signal was periodically recorded during the spin‐coating and annealing stages.

In the control film, a clear PL signal appeared immediately after the antisolvent was dripped at the 25th s of spin‐coating. However, in the target film, the PL signal emerged only after 32 s, indicating that AC slows down the nucleation process. It was observed that the PL peak signal in target films initially appeared at a lower wavelength (**690 nm**) than in control films (**701 nm**), which further gradually red‐shifted while the PL peak position remained nearly unchanged in target films during the remaining spin‐coating stage. This suggests that, in control films, nucleation begins with Br‐rich regions, followed by the diffusion of I^−^ ions into these sites. However, in the target films, AC inhibits Br‐rich nucleation and instead promotes the formation of a stable higher bandgap Cl‐rich perovskite. The preferential nucleation of the Cl‐rich nuclei can be attributed to Pb^2+^ ions forming stronger bonds with Cl^−^ than Br^−^ and I^−^ in DMF:DMSO solvent.

**Figure 3:**
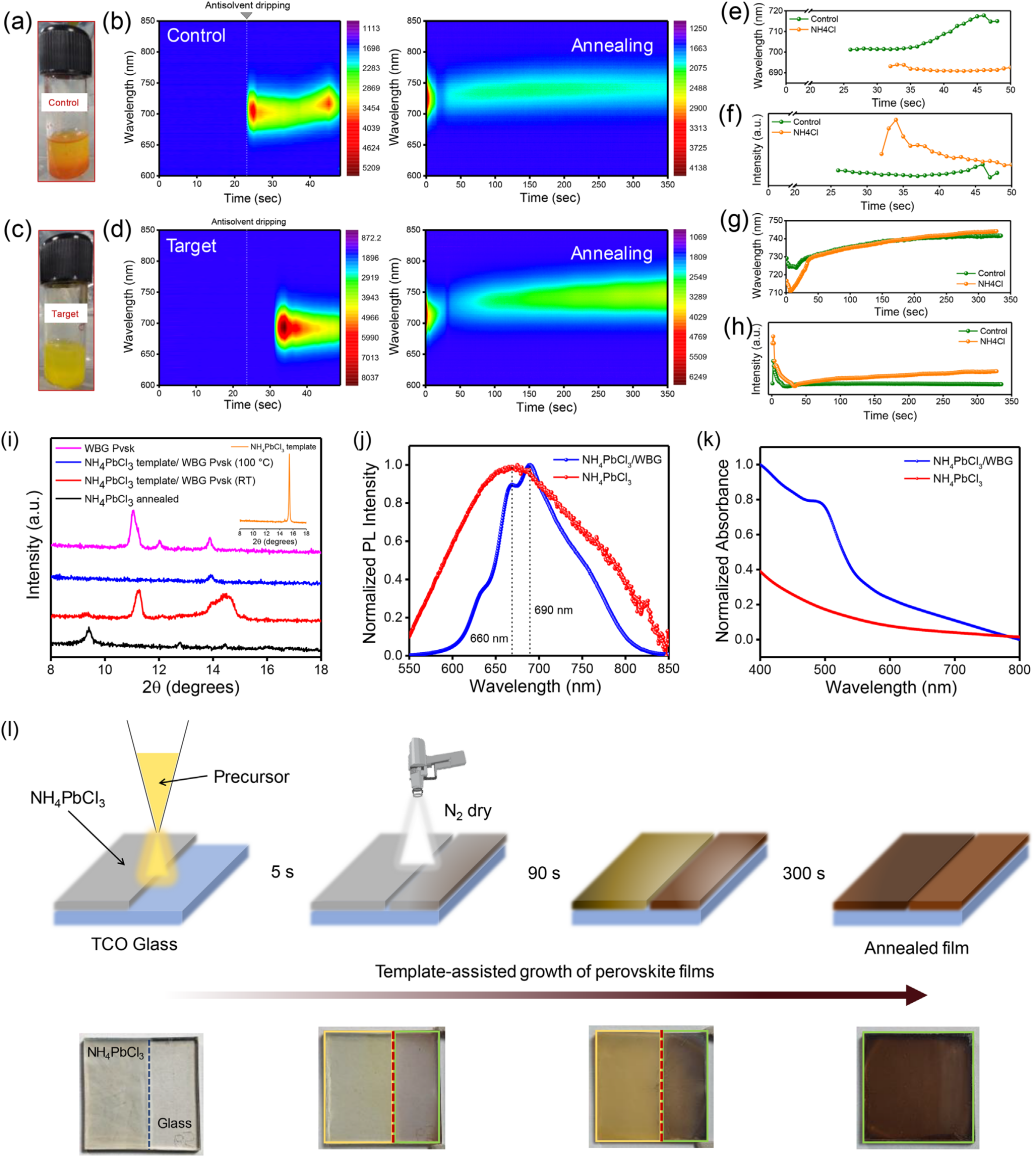
a,b) Photographs of control and target WBG precursor after supersaturation depicting the dominant nucleating species. In situ PL intensity maps as a function of time of the c) control and d) target perovskite films during the spin‐coating and annealing stages. PL data was taken with a rate of one scan per second. e,f) The rate of change of control and target perovskite film's peak wavelength and intensity during the spin‐coating stage. g,h) The rate of change of control and target perovskite film's peak wavelength and intensity during the annealing stage. i–k) Template‐assisted growth of WBG perovskite films is depicted through (i) XRD patterns, (j) PL spectra, and (k) absorbance spectra of the perovskite films. l) Schematic of the template‐assisted growth process of perovskite films.

The change in the initial crystallization pathways with the addition of AC is further verified through the dropwise addition of antisolvent (anisole) to the perovskite precursor solutions, triggering the precipitation of either intermediates or different perovskite phases (**Figure** **3**a,b). Photographs of these perovskite precursors at different time intervals after antisolvent addition (Figure S8, Supporting Information) show that the control solution precipitated into an orange colored solid, indicating Br‐rich perovskite formation, while the target solution yielded a bright yellow precipitate, signifying Cl‐rich perovskite. These observations suggest that AC facilitates the nucleation of Cl‐containing intermediate‐phase nuclei from the precursor solution. Therefore, the perovskite formation likely proceeds through the formation of lead‐halide pre‐nucleated clusters in the solution.

Next, the PL intensity increased rapidly in the target film upon antisolvent quenching due to the rapid nucleation of Cl‐rich perovskite nuclei, which was accompanied by stronger PL intensity, suggesting the formation of several larger‐sized perovskite crystals leading to a shift in PL peak position. Notably, the PL intensity significantly reduced from its peak value at ≈35 s, which can be attributed to the presence of non‐radiative hexagonal polytype phases and the dissolution of perovskite nuclei on the surface during antisolvent‐induced solvent extraction. Interestingly, the PL intensity in the control film is lower than in the target films, owing to the greater fraction of photoinactive hexagonal polytype phases, which might introduce lattice strain‐induced defects.

The PL evolution in the control films takes place in two stages. Initially, there is a rapid increase in the PL intensity after antisolvent dripping, reaching a maximum at 28 s, owing to the supersaturation‐induced nucleation of Br‐rich nuclei. This is followed by a gradual decrease in PL intensity as nuclei redissolve and photoinactive intermediate hexagonal polytype phases are formed. After 35 s, the PL intensity began to increase again, indicating the formation of I‐diffused Br‐rich perovskite crystals. The samples were transferred onto a hotplate for annealing at 100 °C, and the PL intensity was tracked as a function of time, as shown in Figure 3c,d. The evolution of PL emission peaks during the annealing process followed three separate stages, i.e., secondary nucleation, ion‐diffusion, and crystal growth in both the films, before reaching phase stabilization. The annealing process provides enough thermal energy to initiate the solvent evaporation and trigger secondary nucleation. This leads to an increase in the density of smaller nuclei, causing a blueshift in the PL peak position. In the target films, the PL peak position blue shifted from 716 to 710 nm with increased PL intensity up to 7 s, indicating the formation of additional Cl‐rich nucleation sites. Similarly, in the control films, the PL peak shifted from 729 to 723 nm, with increased PL intensity for 16 s, indicating secondary nucleation of Br‐rich nuclei centers. During the second stage, I^−^ ions rapidly diffused into the respective Br‐ and Cl‐rich perovskite nuclei clusters due to the elevated temperature, causing a redshift in the PL peak position for ≈33 s in the target films and 22 s in the control films, before reaching the I^−^ and Br^−^ crystal growth stage. The delayed onset of the crystallization suggests that AC retards the crystal growth stage in target films owing to the increased activation energy barrier for perovskite growth.

As crystallization progressed, the PL intensity of both the control and target films increased, indicating further crystallization of the perovskite films. In the control films, the PL peak intensity saturated at a constant position after ≈278 s, indicating the formation of a stable I/Br phase of WBG perovskite. However, the target films showed a stronger and continuously increasing PL intensity, suggesting the formation of highly crystalline, larger or abundant perovskite crystals, along with in situ elimination of stacking defects. This defect suppression suggests that AC assists in achieving balanced crystallization kinetics and releases lattice strain during crystallization. Further, the target perovskite film exhibited narrower distribution in the FWHM values of the PL peaks during the spin‐coating and annealing stages, indicating a more uniform size distribution of luminescent moieties (Figure S9, Supporting Information). In the target films, the FWHM values stabilized after 300 s during annealing, whereas they continued to increase in the control films. The time evolution of the PL peaks for both films during the spin‐coating and annealing stages is shown in Figure S10 (Supporting Information).

To gain insight into the mechanism of perovskite formation with and without the AC additive, we collected time‐dependent optical images during template‐assisted growth, as shown in Figure 3l. Due to the higher diffusivity of NH_4_^+^ compared to other cations in WBG perovskite and faster nucleation of Cl‐rich perovskite, we used NH_4_PbCl_3_ as a template to monitor perovskite formation. To do so, we partially deposited NH_4_^+^‐based Cl‐rich perovskite (NH_4_PbCl_3_) thin film on half of a glass substrate (region i) while leaving the other half bare (region ii). Next, we deposited WBG perovskite precursor solution on the entire substrate by spin coating, followed by N_2_ drying. Within 5 s, the bare region (ii) spontaneously turned light brown from the outer edges, progressing to a dark brown color in 90 s. However, the middle region at the intersection of regions i and ii remained partially yellow. Interestingly, region i exhibited sluggish transformation behavior with yellow color persisting even after 90 s of drying. This suggests that NH_4_PbCl_3_ delays the cation exchange process and slows down the nucleation rate of lead halide [PbI_2_(Br_2_)] and organic‐inorganic halide salt (FAI/CsI). Such a process slowed down the crystal growth, eventually leading to high‐quality perovskite films, as evident from the intense dark brown annealed film obtained compared to films grown on the bare region.

To further analyze the impact of the NH_4_^+^‐based Cl‐rich perovskite template, we characterized the films using XRD, PL and absorbance measurements. As illustrated in Figure 3j, the as‐deposited NH_4_PbCl_3_ film exhibited strong PL emission at 660 nm, while the WBG perovskite grown on top of NH_4_PbCl_3_ showed an additional emission peak at ≈690 nm, corresponding to the initial perovskite phase observed in in situ PL measurements. These results indicate that NH_4_^+^‐based Cl‐rich perovskite serves as a template for the nucleation of WBG perovskite by mediating the cation exchange process between NH_4_^+^ and FA^+^/Cs^+^, thereby slowing down crystal growth. The UV absorption spectra of NH_4_PbCl_3_ films before and after the cation exchange process are shown in Figure 3k. After the reaction, the absorption increased and extended across the entire visible spectral range with the absorption onset red shifting to ≈550 nm. The templating effect was further confirmed by XRD results (Figure 3i), which showed diffraction peaks at ≈14° corresponding to WBG perovskite absorber, along with a shoulder peak appearing at ≈15° ascribed to NH_4_^+^‐based Cl‐rich perovskite. After annealing, the peak associated with NH_4_^+^‐based Cl‐rich perovskite completely disappeared, likely due to the loss of volatile NH_3_ and HI (HCl) as a byproduct, which further facilitated phase transformation.

#### 2.3.2 Nucleation and Growth Kinetics Study

Based on the observations from colloidal and coordination chemistry, as well as in situ PL and in situ GIXRD studies, we propose a relationship between nucleation‐growth kinetics, stacking defects, and phase transition behavior as schematically shown in Figure S11 (Supporting Information). The process of transformation of perovskite ink into the 3C phase is mediated by the disordered colloids present in the perovskite precursor solution. However, strong interactions between perovskite precursors and DMSO hinder perovskite crystal growth, preventing the complete transformation from the precursor phase to the 3C phase. The strong complexation of DMSO with Pb^2+^ centers in PbI_2_ leads to the formation of corner‐sharing PbI_2_(DMSO)_2_ or edge‐sharing PbI_2_(DMSO) species in colloidal solution **(Step I)**. These iodoplumbate(DMSO)_x_ complexes increase the kinetic barrier for their rearrangement into high valent iodide‐coordinated iodoplumbate motifs such as PbI_5_ or PbI_6_, which are essential for the formation of the final perovskite lattice. Consequently, in control films, the phase conversion pathway proceeds via kinetically governed competitive crystallization between multiple polytype phases (2H, 4H, and 6H) and the 3C phase as they nucleate from the intermediate adduct (sol–gel state) phase, thereby introducing stacking defects within the growing perovskite lattice during competitive crystallization (**Steps II and III**). However, in the presence of AC, DMSO molecules de‐coordinate from iodoplumbate(DMSO)_x_ complex due to the strong donor capability of Cl relative to DMSO, forming strong Pb‐Cl bonds. Reduced coordination of DMSO further promotes the connection of competing halide ions from other iodoplumbate molecules with central Pb^2+^, leading to the formation of higher‐valent polyhalide plumbates. Consequently, this process facilitates the initial stage of breaking the PbI_2_ framework, resulting in the formation of mixed polyhedral PbI_n_Br_1‐n_Cl_1‐n_ complexes (**Step IV**). In the subsequent step, the process of migration and reorganization of polyhedra is facilitated, promoting the vertical alignment and connectivity for perovskite formation (**Step V**). However, a strong Pb‐Cl bond renders expulsion of Cl^−^, a thermodynamically less favorable process, leading to nucleation of intermediate chloride‐rich 3C perovskite characterized by lower formation energy, overall leading to slower crystal growth.

We further postulate that this intermediate perovskite phase proceeds through preferential diffusion of smaller NH_4_^+^ cations (ionic radius of NH_4_^+^: 1.61 Å, FA^+^: 2.79 Å, Cs^+^: 1.73 Å) into the mixed octahedra layers. Owing to its smaller size and higher diffusivity compared to FA^+^ and Cs^+^, NH_4_^+^ readily intercalates into the perovskite lattice during the early stages of nucleation.^[^ ^45^ ^]^ This NH_4_^+^‐rich intermediate phase undergoes cation exchange with FA^+^ and Cs^+^ accompanied by simultaneous diffusion of halide ions during subsequent stages of crystallization.^[^ ^50^ ^]^ This intermediate phase acts as a template, providing extra heterogeneous nucleation sites that compensate for the insufficient nucleation of perovskite caused by deficient CsI and FAI precipitation. As a result, the templated growth mechanism governs the suppression of competing polytype phases by stabilizing the 3C phase and regulating the ion migration pathways.^[^ ^51^, ^52^ ^]^ We propose that the AC‐induced cation exchange process, along with inhibited gel state formation, regulates the diffusion rate of FA^+^ and Cs^+^ into the mixed polyhedra, thereby slowing down the crystal growth, ultimately leading to better crystal quality of perovskite films with suppressed phase segregation and enlarged grain size (discussed in the later section).

From the above analysis, we conclude that AC additive changes the dynamics of the conversion pathway to perovskite precursor →chloride‐rich 3C perovskite phase → polytype phase→ 3C phase. This altered pathway suppresses the 2H/6H phases, which would otherwise transform into the 3C phase. In contrast, the strong perovskite precursor‐solvent interactions in control films result in unbalanced nucleation‐growth kinetics, leading to the simultaneous growth of competing polytype and 3C phases during annealing. By modifying the conversion pathway of WBG perovskites and balancing nucleation‐growth kinetics, AC reduces in situ stacking defects and microstrain in target perovskite films. These findings highlight the crucial role of AC in altering the conversion pathway of WBG perovskites and enabling in situ defect elimination. Notably, our work is the first to report suppression of polytype formation in WBG perovskites through a volatile ammonium additive in DMF/DMSO, as supported by in situ characterizations during spin coating and thermal annealing. Interestingly, our work strikingly differs from the earlier studies that reported the beneficial role of long‐chain chloride‐based additives in promoting the formation of the hexagonal intermediate in WBG perovskites.

## 3 Film and Device Characterizations

### 3.1 Film Characterizations

**Figure 4:**
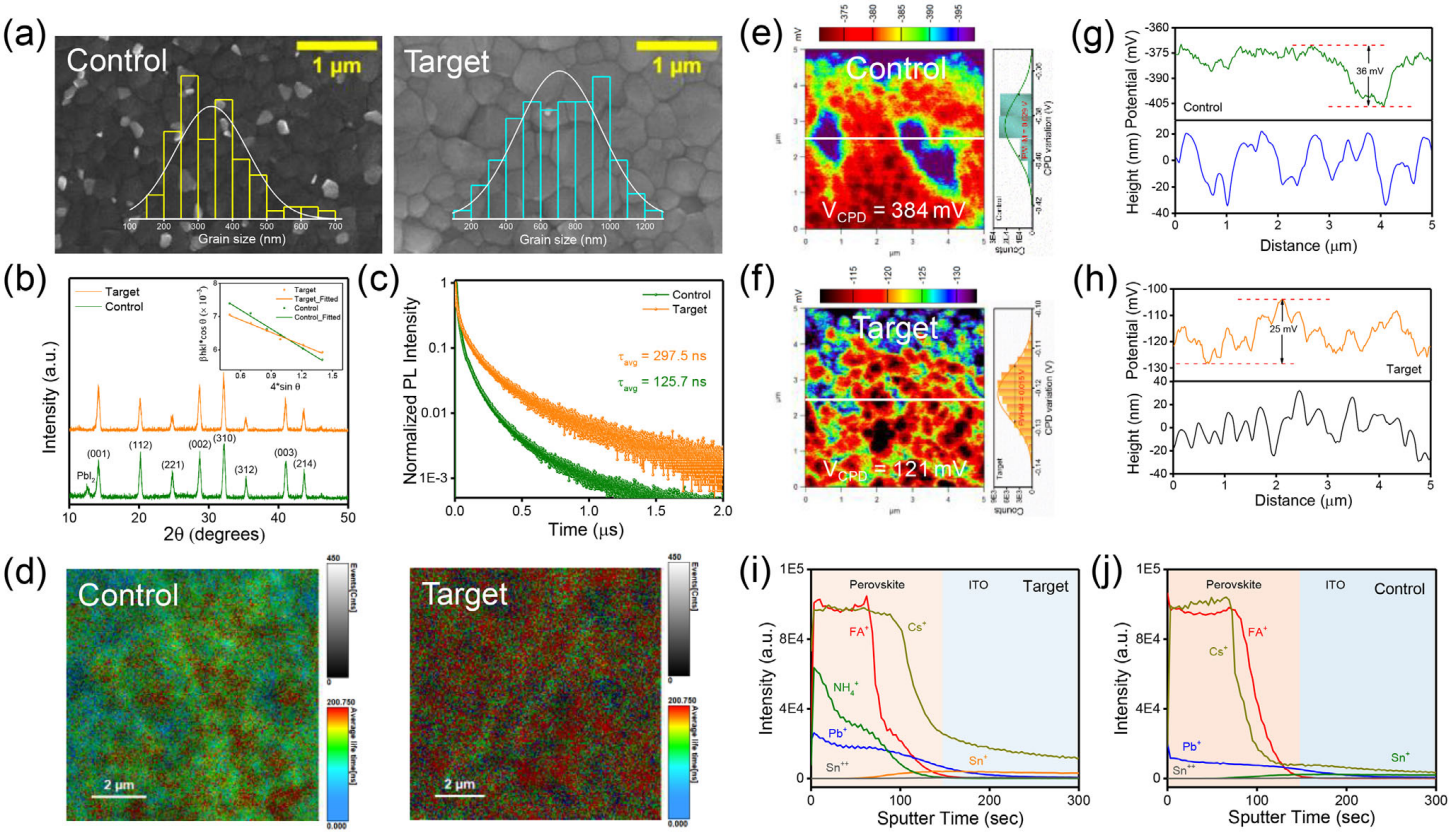
a) Top‐view FESEM micrographs of the control and target perovskite films. b) X‐ray diffraction pattern of the as‐annealed control and target perovskite films. Inset: Williamson‐Hall plot depicting the compressive strain present in the perovskite films. c) TRPL spectra depicting the average lifetime of carriers in control and target perovskite films. d) PL lifetime mapping of control and target perovskite films. e,f) KPFM surface potential maps of the control and target perovskite films. Corresponding height profile of the g) control and h) target perovskite films. ToF‐SIMS depth profiles showing cation distribution in i) target and j) control WBG perovskite films.

We investigated the influence of the conversion pathways on the properties of the perovskite films through a series of characterizations. The scanning electron microscopy (SEM) micrographs (**Figure** **4**a) revealed increased average grain size from 338±105 to 708 ± 241 nm upon AC addition, along with an increase in the root mean square roughness (RMS) from 26 to 33 nm as evident from the atom force microscopy (AFM) height images (Figure S12, Supporting Information). The improvement in the grain quality can be ascribed to chlorine (Cl) incorporation, which slows down the crystal growth of perovskite, thus leading to balanced nucleation–growth kinetics. The SEM micrographs for different concentrations of AC additive in the perovskite precursor are depicted in Figure S13 (Supporting Information). The X‐ray diffraction (XRD) measurements shown in Figure 4b revealed that both perovskite films have a phase‐pure cubic crystal structure along with a small amount of residual PbI_2_ (2θ = 12.6°). Meanwhile, AC suppressed the intensity of the unreacted PbI_2_ peak. AC‐based perovskite film exhibited a stronger intensity of the characteristic (001) diffraction peak, suggesting AC aids in improving crystallinity. In the target films, the average crystallite size increased from 47.6 to 53.4 nm, while the FWHM of (001) diffraction peak decreased from 0.42° to 0.37°. Additionally, compared to the control films, the cubic (003) and (214) diffraction peaks in the planes shifted to higher angles (Figure S14, Supporting Information). This shift indicates a decrease in out‐of‐plane lattice parameters and relaxation in‐plane macrostrain, confirming the incorporation of smaller Cl^−^ ions into the perovskite lattice due to stronger Pb─Cl bonding.

Further, we investigated the microstrain in the out‐of‐plane orientation of perovskite films using the Williamson–Hall (WH) method (Inset of Figure 4b). The microstrain (ε) was calculated from the slope obtained by linearly fitting the plot of *β_hkl_ cosθ* versus *4sinθ*. The target films exhibited a lower strain of 1.26 × 10^−3^ compared to control films (1.89 × 10^−3^), which can be attributed to improved crystallization. Alternatively, according to Poisson's equation, this also indicates that AC addition reduces compressive strain in the out‐of‐plane direction and relaxes strain in the in‐plane direction. The XRD patterns of the perovskite films with different concentrations of AC additive are shown in Figure S15 (Supporting Information).

The effect of AC on the optoelectronic properties of perovskite films is shown by the absorption and PL spectra of the control and target films, as shown in Figure S16 (Supporting Information). The target films exhibited noticeably higher absorbance across the visible spectrum compared to the control samples, indicating stronger light absorption. The Tauc plot depicting the optical bandgap of control and target samples is shown in Figure S17 (Supporting Information). The target films exhibited higher steady‐state (SS) PL intensity than the control films, indicating reduced defect states. This suggests suppressed non‐radiative recombination, likely due to improved crystallization kinetics and reduced residual strain. Notably, the SSPL spectra of the control films revealed phase separation between Br‐rich and I‐rich perovskite domains, with a blueshift in the PL peak indicating the formation of the Br‐rich phase. In contrast, the target films showed a red‐shifted PL peak and a narrower FWHM than the control films, suggesting a more homogeneous phase distribution and reduced halide segregation. The PL spectra of perovskite films with different concentrations of AC additive concentrations are shown in Figure S18 (Supporting Information).

To further analyze charge carrier dynamics, we measured the time‐resolved photoluminescence (TRPL) response of the control and target films deposited on a glass substrate. The PL decay curves revealed a significantly longer average carrier lifetime of 297.5 ns in the target films compared to 125.7 ns in the control sample. This indicates fewer non‐radiative pathways in the target films, leading to more efficient charge carrier transport and a corresponding increase in *V_oc_* in the device (Figure 4c). The lifetime decay parameters obtained from TRPL spectra fitting are provided in Table S2 (Supporting Information). As shown in Figure 4d, fluorescence lifetime mapping of the films revealed a uniform carrier emission rate across the entire measured area (2 × 2 µm^2^) in the target films. In contrast, the control films suffered from significant nonradiative recombination, characterized by regions with lower lifetimes due to greater heterogeneities in the films.

X‐ray photoelectron spectroscopy (XPS) spectra of the control and target films are shown in Figure S19 (Supporting Information). In the AC‐based perovskite film, the peaks corresponding to Pb 4f, I 5d, and Br 3d shifted to lower binding energies compared to the control film. This shift is attributed to lattice contraction and the redistribution of electron cloud density.^[^ ^53^ ^]^ It suggests that Cl is incorporated at the I/Br sites in the wide‐bandgap (WBG) perovskite lattice, leading to lattice contraction. To further analyze the electrical properties of perovskite films, we performed Kelvin probe force microscopy (KPFM) measurements. As shown in Figure 4e,f, the AC‐based perovskite film showed a more uniform surface potential (SP) distribution than the pristine film. The SP value decreased from 384 to 121 mV, indicating a stronger n‐type character in the AC‐based perovskite film, which enhances charge extraction and transport efficiency.^[^ ^54^ ^]^ The control films exhibited a larger *V_CPD_* variation between GBs and adjacent GI (Figure 4g,h). This could be attributed to higher strain in the control films that promotes the migration of halide ions toward the GBs to compensate the space charge, thereby significantly lowering *V_CPD_* at GBs.^[^ ^55^ ^]^ The accumulation of halide ions at the grain boundaries traps charge carriers, leading to halide segregation and the appearance of darker regions/clusters in the surface potential map. In contrast, the target films showed a nearly uniform *V_CPD_* between GBs and GI, attributed to lower lattice strain that increases the barrier for halide ion migration and decreases the defect density. Further, conductive atomic force microscopy (CAFM) measurements revealed a reduction in the dark current at GBs in target films (Figure S20, Supporting Information), associated with a reduction in halide ion migration at the GBs.

Previous studies have demonstrated that a low boiling point solvent system shortens sol–gel duration time, thereby suppressing the cation diffusion, which could effectively inhibit phase segregation in perovskite films.^[^ ^46^ ^]^ Halide‐cation spatial distribution within the perovskite layer was chemically analysed by time‐of‐flight secondary‐ion mass spectroscopy (ToF‐SIMS). The ToF‐SIMS depth profiles in Figure 4i,j exhibits slower decay in intensity of Pb^2+^, FA^+,^ and Cs^+^ along with the presence of NH_4_^+^ in as‐deposited target films compared to control films. This is attributed to a shorter sol–gel state owing to decreased perovskite‐solvent coordination. Further, it also indicates that the presence of NH_4_^+^ lowers the diffusivity difference among cationic species, leading to less significant compositional fluctuation (cations) in target sample than that of the control one. This finding is consistent with the previous reports, which demonstrated that the diffusivity difference of cationic pairs guides phase segregation. Therefore, we suggest that by weakening perovskite‐solvent interaction and selecting suitable A‐site cationic pairs, it is possible to efficiently suppress phase segregation across the thickness of WBG perovskite film. The negative mode of ToF‐SIMS revealed a uniform distribution of I^−^ and Br^−^ in both as‐deposited control and target films, as depicted in Figure S21a (Supporting Information). However, as compared to control films (Figure S21b, Supporting Information), annealing of target films caused a significant redistribution‐ the intensities of I^−^ and Br^−^ increased in the bulk while decreasing more substantially near the surface (Figure S21c, Supporting Information). This suggests that these ions diffuse into the bulk during the annealing process. In the target film, after AC dissociation, the Cl^−^ ions migrated through the bulk and accumulated at the buried interface (Figure S21d, Supporting Information). This accumulation promotes oriented crystallization of the perovskite, which is expected to improve the photostability of the target PSCs.^[^ ^56^, ^57^ ^]^ Moreover, the resulting halide gradient is likely to induce an additional built‐in electric field, improving charge extraction and enhancing the photovoltaic performance of the target devices.^[^ ^58^ ^]^

### 3.2 Defects Analyses

**Figure 5:**
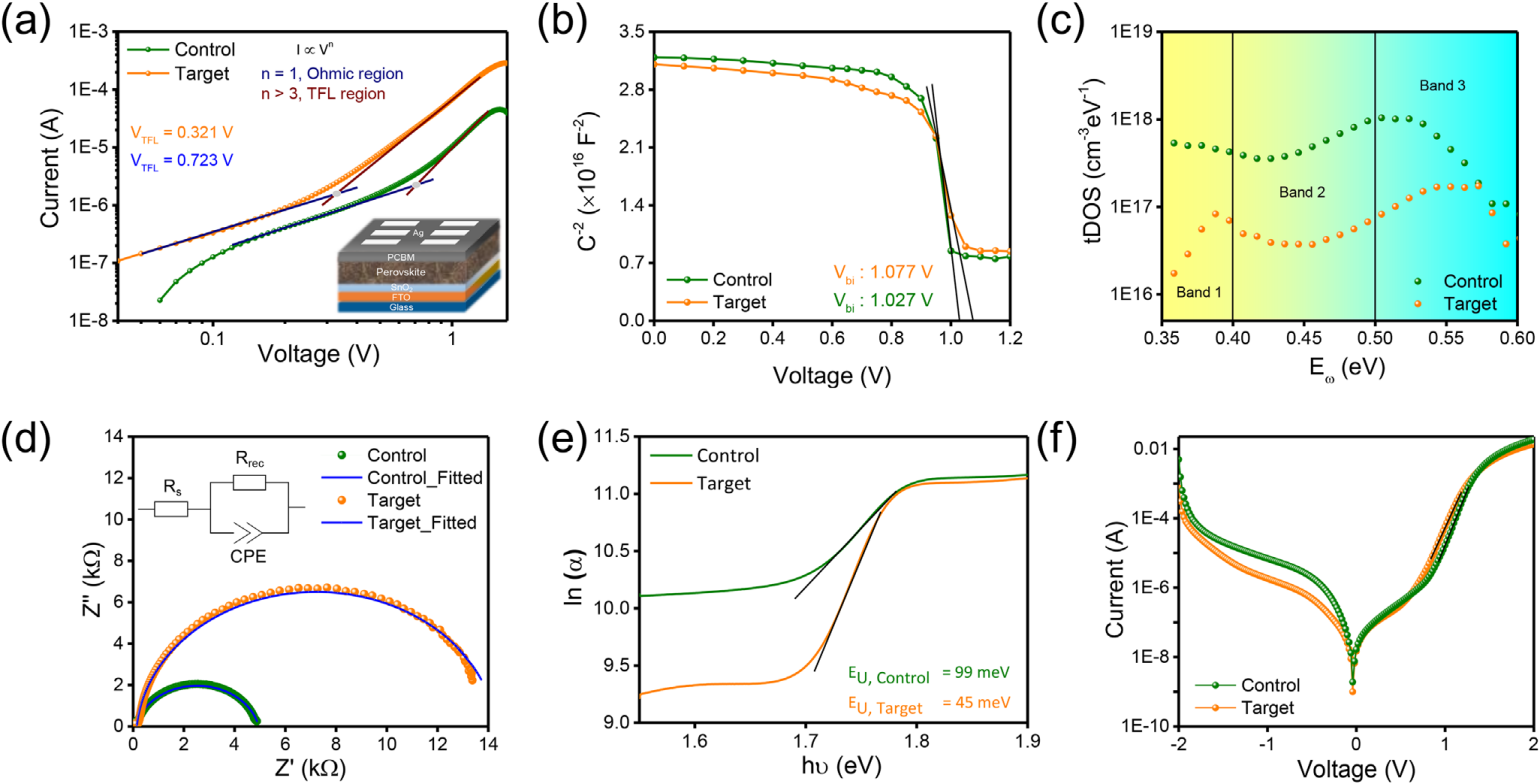
a) Dark *I*–*V* curves of the electron‐only device (FTO/ SnO_2_/ Perovskite/ phenyl‐C_61_‐butyric acid methyl ester (PCBM)/ Ag) based on the control and target perovskite films (Scan range: −2 to 2 V with a step size of 0.02 Vs^−1^). b) Mott–Schottky curves of the control and target perovskite devices. c) t‐DOS spectra and d) Electrical impedance spectra of the control and target perovskite devices. e) Urbach energy tail depicting the degree of electronic disorder in the control and target perovskite films as obtained from the absorbance data. f) Dark *I*–*V* curves of the control and target perovskite devices.

We estimated the trap density (*N_trap_*) of the films using the space charge limited current (SCLC) technique. It was calculated using the equation

(1) $$N_{trap}=\left(2\varepsilon \varepsilon_{o}V_{TFL}\right)/\left(eL^{2}\right)$$

where *ε* is the relative permittivity, *ε_o_* is the vacuum dielectric constant, *V_TFL_* is the trap‐field limited voltage, *e* is the elemental charge, and *L* is the perovskite layer thickness. As shown in the dark *J–V* curves (**Figure** **5**a) of the electron‐only device, the target film exhibited a lower *N_trap_* of 4.32 × 10^15^ cm^−3^ as compared to 1.04 × 10^16^ cm^−3^ of the control film. This reduction can be attributed to the self‐elimination of stacking defects during solution processing and improved crystallographic orientation. To analyse built‐in potentials (*V_bi_*) in the perovskite layers, we performed Mott–Schottky measurements. The target film showed a higher *V_bi_* of 1.077 V compared to 1.027 V in the control film (Figure 5b). This suggests a more efficient extraction of photogenerated charge carriers, leading to a higher *V_oc_* in AC‐based devices, as discussed in the later section.

Next, the thermal admittance spectroscopy was utilized to determine the energetic distribution of trap states in perovskite films. Figure 5c shows that AC‐treated films had a reduced trap density of states, both at shallow and deep energy levels. The reduction in shallow‐level defects could be attributed to a more homogeneous phase distribution, while the presence of Cl at the interface between perovskite and electron transport layers, along with the elimination of stacking defects and strain, helps suppress metal‐related deep‐level defects. To investigate interfacial charge transport and recombination behavior, we conducted electrochemical impedance spectroscopy (EIS). Figure 5d shows Nyquist plots of control and target devices at an applied voltage of 0.9 V. The target devices demonstrated a lower charge transfer resistance (*R_con_* = 0.094 Ohm.cm^−2^) and higher recombination resistance (*R_rec_* = 14.16 Ohm.cm^−2^) compared to the control device (*R_con_* = 127.92 Ohm.cm^−2^, and *R_rec =_* 4.85 Ohm.cm^−2^). This indicates a more effective charge transfer and a strong barrier to recombination, ultimately leading to improved device performance. The parameters obtained after fitting Nyquist plots using an equivalent circuit model are given in Table S3 (Supporting Information). These results suggest that modifying the conversion pathways by preventing the formation of hexagonal intermediate phases helps reduce defects and lower device resistance.

To further analyse the band‐edge defect states, we examined the Urbach tail profiles using the equation α = α_o_ exp(hʋ/E_U_) (α: absorption coefficient, α_o_: constant, and *E_U_*: Urbach energy). The target perovskite films exhibited a lower *E_U_* of 45 meV compared to the 99 meV in the control films, indicating reduced electronic disorder at the band edge, which helps in minimizing the voltage deficits (Figure 5e). The reduced disorder in target films could be attributed to the role of AC in promoting the formation of the 3C phase and suppressing non‐perovskite hexagonal polytype phases. This results in more uniform grain growth, enhanced crystallinity, and fewer defect states, ultimately improving material quality and device performance. The dark *J*–*V* curves of the devices, as shown in Figure 5f, show that the target device exhibited a smaller reverse saturation current density (*J_o_*)‐ lower by one order of magnitude compared to the control device. A smaller *J_o_* indicates suppressed trap‐assisted SRH recombination in AC‐based devices. This is further corroborated by the greater slope and smaller ideality factor in the target devices obtained by linearly fitting the dark *J*–*V* data using the diode equation

(2) $$V_{oc}=\left(nk_{B}T/q\right)\;\ln\left[\left(J_{ph}/J_{o}\right)+1\right)]$$

where *n*, *k_B_*, *q*, *T*, *J_ph,_* and *J_o_* denote ideality factor, Boltzmann constant, elementary charge, thermodynamic temperature, photocurrent density, and dark reverse saturation current, respectively.

### 3.3 Device Performance and Photostability

**Figure 6:**
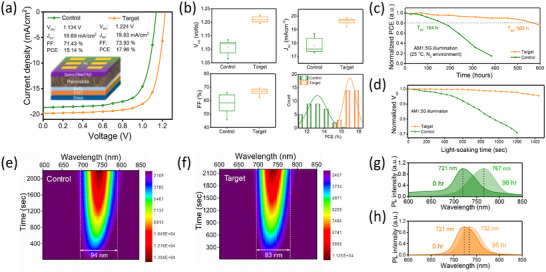
a) *J*–*V* curves of the control and target perovskite solar cells in the n‐i‐p architecture. b) The statistical distribution of photovoltaic parameters (V_oc_, J_sc_, FF, and PCE) of control and target perovskite solar cells. c) The long‐term light stability of the unencapsulated control and target perovskite solar cells was measured under AM1.5G illumination (Xe lamp) in an N_2_ environment. d) Open‐circuit voltage tracking of control and target perovskite solar cells under prolonged light exposure. In situ PL maps of the e) control and f) target perovskite films measured under AM1.5G illumination for 40 min. Corresponding PL spectra of the g) control and h) target perovskite films showing the PL peak shift under light exposure.

To demonstrate the impact of modulating perovskite‐solvent interaction on device performance, we fabricated n‐i‐p structured WBG PSCs with the architecture FTO/SnO_2_/perovskite/Spiro‐OMeTAD/Au. Figure S22 (Supporting Information) presents the *J*–*V* characteristics (under AM1.5 illumination) at different AC concentrations, while the *J–V* parameters are summarized in Figure S23 and Table S4 (Supporting Information). The control device showed a champion PCE of 15.14% with *V_oc_* of 1.13 V, short‐circuit density (*J_sc_*) of 18.69 mA cm ^−2^ and fill factor (FF) of 71.43%. In contrast, the AC‐based device outperformed the control by showing a champion PCE of 17.96% with an improved *V_oc_* of 1.22 V, *J_sc_* of 19.83 mA cm^−2^ and FF of 73.93% (**Figure** **6**a). We calculated a *J_sc_* of 18.89 mA cm^−2^ from the EQE spectrum shown in Figure S24 (Supporting Information). Additionally, the target devices showed lesser hysteresis between reverse and forward scans (Figure S25, Supporting Information). Figure 6b shows the statistical distribution of key photovoltaic (PV) parameters (PCE, *V_oc_*, *J_sc,_* and FF) for 30 devices (15 devices per condition), demonstrating high reproducibility in target devices. The narrower PCE distribution in target devices can be attributed to the improvement in all the PV parameters. The enhancement in the *V_oc_* and FF can be attributed to improvements in the perovskite thin film quality, such as increased crystallinity, reduced microstrain, and reduction in the stacking defects. The performance of target devices in this work is comparable to various chloride additives reported in literature for wide‐bandgap perovskite solar cells (Table S5, Supporting Information).

To evaluate photostability, we tested both control and target devices at 25 °C under continuous AM1.5G light illumination in an N_2_ environment. As shown in Figure 6c, the target devices demonstrated significantly improved photostability and retained 80% of their initial PCE for more than 550 h. In contrast, the control devices degraded much faster, exhibiting a sharp decline in the PCE, highlighting their poor photostability. One of the major challenges in WBG PSCs with increased Br content is *V_oc_* loss due to halide demixing. To investigate this, we monitored the *V_oc_* evolution in control and target devices for 25 min, as shown in Figure 6d. While the control devices exhibited a gradual *V_oc_* decline, indicating a high degree of halide‐segregation, the target devices maintained a stable *V_oc_*, suggesting the halide segregation is suppressed upon incorporating the AC additive.

To gain insights into the origin of *V_oc_* loss, we conducted in situ PL measurements on the films, as shown in Figure 6e,f. The control films showed a redshift in the PL peak after 96 h of continuous light exposure. Furthermore, the increase in FWHM and decrease in PL intensity suggest a higher level of non‐radiative recombination, typically caused by the presence of defects. These defects act as a driving force for phase segregation, leading to the formation of iodide‐ and bromide‐rich domains in WBG perovskite films. In contrast, the target films maintained a consistent PL peak position, FWHM, and PL intensity, as shown in Figure 6g,h. indicating suppressed phase separation. This is attributed to the improved perovskite film quality after incorporating the AC additive, which reduced stacking defects and lowered microstrain. Further evidence of improved structural order after AC incorporation is observed through the Urbach energies calculated from the time‐dependent absorption spectra of the perovskite films (see Figure S26 and Table S6, Supporting Information). The target films displayed lower electronic disorder as compared to the control films.

In summary, several key factors contribute to suppressed halide segregation in AC‐based films. First, the in situ suppression of stacking defects reduces microstrain and preserves the structural stability of the perovskite crystal lattice. Second, the controlled crystallization process minimizes the formation of I‐rich phases, which would otherwise serve as nucleation centers for photoinduced halide demixing.^[^ ^25^, ^59^, ^60^ ^]^ Further, the presence of multiple halides (Cl, Br, and I) as confirmed from Wavelength Dispersive X‐Ray Fluorescence (WD‐XRF) (see Figure S27 and Table S7, Supporting Information) and the TOF‐SIMS measurements, increases the activation energy barrier for the phase separation, which subsequently homogenizes the mixed halide phase.^[^ ^61^ ^]^

## 4 Conclusion

In this work, we studied the impact of volatile ammonium chloride (AC) additive on the phase formation dynamics of WBG halide perovskite using in situ GIXRD and PL measurements. We correlated the perovskite formation mechanism with the morphological, structural, and optoelectronic properties and highlighted its beneficial effects on WBG perovskite performance. The incorporation of AC alongside DMSO as a solvent weakened the solvent‐perovskite precursor coordination, leading to more balanced nucleation‐growth kinetics and reduced microstrain, lowering the in situ defect density. By modulating the solvent‐perovskite interactions, AC altered the crystallization pathway, suppressing the formation of intermediate complexes and enabling the formation of phase‐pure perovskite films with higher crystallinity and compositional homogeneity. Moreover, AC‐based perovskite films exhibited improved optoelectronic and structural properties, such as increased grain size, fewer defects, and reduced lattice microstrain. As a result, devices utilizing these modified films exhibited better photovoltaic performance, with a PCE of ≈18%, along with greater photostability. This work provides a comprehensive understanding of solvent‐perovskite coordination chemistry and introduces a strategic additive engineering approach to optimize these interactions and tailor the crystallization process. This method enables the formation of high‐quality perovskite films with enhanced properties, paving the way for the development of high‐performance, photostable WBG PSCs.

## Conflict of Interest

The authors declare no conflict of interest.

## Supporting information



Supporting Information

## Data Availability

The data that support the findings of this study are available from the corresponding author upon reasonable request.

## References

[1] R. D. J. Oliver , P. Caprioglio , F. Peña‐Camargo , L. R. V. Buizza , F. Zu , A. J. Ramadan , S. G. Motti , S. Mahesh , M. M. McCarthy , J. H. Warby , Y.‐H. Lin , N. Koch , S. Albrecht , L. M. Herz , M. B. Johnston , D. Neher , M. Stolterfoht , H. J. Snaith , Energy Environ. Sci. 2022, 15, 714.

[2] I. Susic , L. Gil‐Escrig , F. Palazon , M. Sessolo , H. J. Bolink , ACS Energy Lett. 2022, 7, 1355. 35434366 10.1021/acsenergylett.2c00304 PMC9004330

[3] G. Kim , C. S. Moon , T.‐Y. Yang , Y. Y. Kim , J. Chung , E. H. Jung , T. J. Shin , N. J. Jeon , H. H. Park , J. Seo , Sol. RRL 2020, 4, 2000033.

[4] R. Ranjan , S. Ranjan , M. Monalisa , K. S. Nalwa , A. Singh , A. Garg , R. K. Gupta , Sol. Energy 2021, 225, 200.

[5] T. C.‐J. Yang , P. Fiala , Q. Jeangros , C. Ballif , Joule 2018, 2, 1421.

[6] J. Xu , C. C. Boyd , Z. J. Yu , A. F. Palmstrom , D. J. Witter , B. W. Larson , R. M. France , J. Werner , S. P. Harvey , E. J. Wolf , W. Weigand , S. Manzoor , M. F. A. M. van Hest , J. J. Berry , J. M. Luther , Z. C. Holman , M. D. McGehee , Science 2020, 367, 1097. 32139537 10.1126/science.aaz5074

[7] D. H. Kim , C. P. Muzzillo , J. Tong , A. F. Palmstrom , B. W. Larson , C. Choi , S. P. Harvey , S. Glynn , J. B. Whitaker , F. Zhang , Z. Li , H. Lu , M. F. A. M. van Hest , J. J. Berry , L. M. Mansfield , Y. Huang , Y. Yan , K. Zhu , Joule 2019, 3, 1734.

[8] S. Zhang , M.‐C. Tang , N. V. Nguyen , T. D. Anthopoulos , C. A. Hacker , ACS Appl. Electron. Mater. 2021, 3, 2277. 10.1021/acsaelm.1c00191 PMC11187822 38903952

[9] J. Kim , M. I. Saidaminov , H. Tan , Y. Zhao , Y. Kim , J. Choi , J. W. Jo , J. Fan , R. Quintero‐Bermudez , Z. Yang , L. N. Quan , M. Wei , O. Voznyy , E. H. Sargent , Adv. Mater. 2018, 30, 1706275. 10.1002/adma.201706275 29441615

[10] S. Gharibzadeh , B. Abdollahi Nejand , M. Jakoby , T. Abzieher , D. Hauschild , S. Moghadamzadeh , J. A. Schwenzer , P. Brenner , R. Schmager , A. A. Haghighirad , L. Weinhardt , U. Lemmer , B. S. Richards , I. A. Howard , U. W. Paetzold , Adv. Energy Mater. 2019, 9, 1803699.

[11] D. B. Khadka , Y. Shirai , M. Yanagida , T. Noda , K. Miyano , ACS Appl. Mater. Interfaces 2018, 10, 22074. 29888594 10.1021/acsami.8b04439

[12] M. Anaya , G. Lozano , M. E. Calvo , H. Míguez , Joule 2017, 1, 769.

[13] E. L. Unger , L. Kegelmann , K. Suchan , D. Sörell , L. Korte , S. Albrecht , J. Mater. Chem. A 2017, 5, 11401.

[14] E. T. Hoke , D. J. Slotcavage , E. R. Dohner , A. R. Bowring , H. I. Karunadasa , M. D. McGehee , Chem. Sci. 2015, 6, 613. 28706629 10.1039/c4sc03141e PMC5491962

[15] Z. Ding , H. Yang , S. Li , D. Wang , Y. Jiang , M. Yuan , ACS Photonics 2024, 11, 5061.

[16] S. Mahesh , J. M. Ball , R. D. J. Oliver , D. P. McMeekin , P. K. Nayak , M. B. Johnston , H. J. Snaith , Energy Environ. Sci. 2020, 13, 258.

[17] Y. An , N. Zhang , Z. Zeng , Y. Cai , W. Jiang , F. Qi , L. Ke , F. R. Lin , S.‐W. Tsang , T. Shi , A. K.‐Y. Jen , H.‐L. Yip , Adv. Mater. 2024, 36, 2306568. 10.1002/adma.202306568 37677058

[18] T. Elmelund , B. Seger , M. Kuno , P. V. Kamat , ACS Energy Lett. 2019, 5, 56.

[19] T. Huang , S. Tan , S. Nuryyeva , I. Yavuz , F. Babbe , Y. Zhao , M. Abdelsamie , M. H. Weber , R. Wang , K. N. Houk , C. M. Sutter‐Fella , Y. Yang , Sci. Adv. 2021, 7, abj1799. 10.1126/sciadv.abj1799 PMC8580316 34757790

[20] Y. Zheng , X. Wu , J. Liang , Z. Zhang , J. Jiang , J. Wang , Y. Huang , C. Tian , L. Wang , Z. Chen , C.‐C. Chen , Adv. Funct. Mater. 2022, 32, 2200431.

[21] P. Gratia , I. Zimmermann , P. Schouwink , J.‐H. Yum , J.‐N. Audinot , K. Sivula , T. Wirtz , M. K. Nazeeruddin , ACS Energy Lett. 2017, 2, 2686.

[22] S. Wang , T. Yang , Y. Yang , Y. Du , W. Huang , L. Cheng , H. Li , P. Wang , Y. Wang , Y. Zhang , C. Ma , P. Liu , G. Zhao , Z. Ding , S. (.F.). Liu , K. Zhao , Adv. Mater. 2023, 35, 2305314. 10.1002/adma.202305314 37652150

[23] K. Wang , M.‐C. Tang , H. X. Dang , R. Munir , D. Barrit , M. De Bastiani , E. Aydin , D.‐M. Smilgies , S. De Wolf , A. Amassian , Adv. Mater. 2019, 31, 1808357. 10.1002/adma.201808357 31206857

[24] M. I. Saidaminov , J. Kim , A. Jain , R. Quintero‐Bermudez , H. Tan , G. Long , F. Tan , A. Johnston , Y. Zhao , O. Voznyy , E. H. Sargent , Nat. Energy 2018, 3, 648.

[25] X. Shen , B. M. Gallant , P. Holzhey , J. A. Smith , K. A. Elmestekawy , Z. Yuan , P. V. G. M. Rathnayake , S. Bernardi , A. Dasgupta , E. Kasparavicius , T. Malinauskas , P. Caprioglio , O. Shargaieva , Y.‐H. Lin , M. M. McCarthy , E. Unger , V. Getautis , A. Widmer‐Cooper , L. M. Herz , H. J. Snaith , Adv. Mater. 2023, 35, 2211742. 10.1002/adma.202211742 37191054

[26] F. Yu , J. Liu , J. Huang , P. Xu , C.‐H. Li , Y.‐X. Zheng , H. Tan , J.‐L. Zuo , Sol. RRL 2022, 6, 2100906.

[27] Y. Chen , N. Yang , G. Zheng , F. Pei , W. Zhou , Y. Zhang , L. Li , Z. Huang , G. Liu , R. Yin , H. Zhou , C. Zhu , T. Song , C. Hu , D. Zheng , Y. Bai , Y. Duan , Y. Ye , Y. Wu , Q. Chen , Science 2024, 385, 554. 39088618 10.1126/science.ado9104

[28] M. Li , R. Sun , J. Chang , J. Dong , Q. Tian , H. Wang , Z. Li , P. Yang , H. Shi , C. Yang , Z. Wu , R. Li , Y. Yang , A. Wang , S. Zhang , F. Wang , W. Huang , T. Qin , Nat. Commun. 2023, 14, 573. 36732540 10.1038/s41467-023-36224-6 PMC9895431

[29] T. Bu , L. K. Ono , J. Li , J. Su , G. Tong , W. Zhang , Y. Liu , J. Zhang , J. Chang , S. Kazaoui , F. Huang , Y.‐B. Cheng , Y. Qi , Nat. Energy 2022, 7, 528.

[30] T. Bu , J. Li , H. Li , C. Tian , J. Su , G. Tong , L. K. Ono , C. Wang , Z. Lin , N. Chai , X.‐L. Zhang , J. Chang , J. Lu , J. Zhong , W. Huang , Y. Qi , Y.‐B. Cheng , F. Huang , Science 2021, 372, 1327. 34140385 10.1126/science.abh1035

[31] Y. Du , Q. Tian , S. Wang , L. Yin , C. Ma , Z. Wang , L. Lang , Y. Yang , K. Zhao , S. (.F.). Liu , Adv. Mater. 2024, 36, 2307583. 10.1002/adma.202307583 37824785

[32] J. Li , R. Munir , Y. Fan , T. Niu , Y. Liu , Y. Zhong , Z. Yang , Y. Tian , B. Liu , J. Sun , D.‐M. Smilgies , S. Thoroddsen , A. Amassian , K. Zhao , S. (.F.). Liu , Joule 2018, 2, 1313.

[33] M. G. Guaita , R. Szostak , F. M. C. da Silva , A. de Morais , R. F. Moral , T. Kodalle , V. C. Teixeira , C. M. Sutter‐Fella , H. C. N. Tolentino , A. F. Nogueira , Adv. Funct. Mater. 2023, 34, 2307104.

[34] D. G. Lee , D. H. Kim , J. M. Lee , B. J. Kim , J. Y. Kim , S. S. Shin , H. S. Jung , Adv. Funct. Mater. 2021, 31, 2006718.

[35] S. J. Lee , J. H. Heo , S. H. Im , ACS Appl. Mater. Interfaces 2020, 12, 8233. 31977182 10.1021/acsami.9b20493

[36] S. Yang , Y. Duan , Z. Liu , S. (.F.). Liu , Adv. Energy Mater. 2023, 13, 2201733.

[37] L. Ke , S. Luo , X. Ren , Y. Yuan , J. Phys. D: Appl. Phys. 2021, 54, 163001.

[38] Y. Deng , X. Zheng , Y. Bai , Q. Wang , J. Zhao , J. Huang , Nat. Energy 2018, 3, 560.

[39] S. Chen , X. Dai , S. Xu , H. Jiao , L. Zhao , J. Huang , Science 2021, 373, 902. 34413234 10.1126/science.abi6323

[40] G. Tong , D.‐Y. Son , L. K. Ono , Y. Liu , Y. Hu , H. Zhang , A. Jamshaid , L. Qiu , Z. Liu , Y. Qi , Adv. Energy Mater. 2021, 11, 2003712.

[41] Y. Tidhar , E. Edri , H. Weissman , D. Zohar , G. Hodes , D. Cahen , B. Rybtchinski , S. Kirmayer , J. Am. Chem. Soc. 2014, 136, 13249. 25171634 10.1021/ja505556s

[42] E. Radicchi , E. Mosconi , F. Elisei , F. Nunzi , F. De Angelis , ACS Appl. Energy Mater. 2019, 2, 3400.

[43] J. Park , S. Kim , Y. H. Chu , J. Lee , D.‐Y. Son , M. Choi , Y. S. Lee , Adv. Energy Mater. 2024, 14, 2400620.

[44] N. Ahn , D.‐Y. Son , I.‐H. Jang , S. M. Kang , M. Choi , N.‐G. Park , J. Am. Chem. Soc. 2015, 137, 8696. 26125203 10.1021/jacs.5b04930

[45] H. Si , Q. Liao , Z. Kang , Y. Ou , J. Meng , Y. Liu , Z. Zhang , Y. Zhang , Adv. Funct. Mater. 2017, 27, 1701804.

[46] L. Liu , Y. Bai , X. Zhang , Y. Shang , C. Wang , H. Wang , C. Zhu , C. Hu , J. Wu , H. Zhou , Y. Li , S. Yang , Z. Ning , Q. Chen , Angew. Chem. 2020, 132, 6035. 10.1002/anie.201914183 31957946

[47] C. Xu , Z. Zhang , S. Zhang , H. Si , S. Ma , W. Fan , Z. Xiong , Q. Liao , A. Sattar , Z. Kang , Y. Zhang , Adv. Funct. Mater. 2021, 31, 2009425.

[48] C. Xu , S. Zhang , W. Fan , F. Cheng , H. Sun , Z. Kang , Y. Zhang , Adv. Mater. 2023, 35, 2207172. 10.1002/adma.202207172 36401565

[49] S. Zhang , C. Xu , W. Fan , H. Sun , F. Cheng , F. Dai , Z. Liang , Z. Kang , Y. Zhang , Matter 2024, 7, 3205.

[50] Z. Zhou , S. Pang , F. Ji , B. Zhang , G. Cui , Chem. Commun. 2016, 52, 3828. 10.1039/c5cc09873d 26867948

[51] J. H. Heo , D. H. Song , H. J. Han , S. Y. Kim , J. H. Kim , D. Kim , H. W. Shin , T. K. Ahn , C. Wolf , T.‐W. Lee , S. H. Im , Adv. Mater. 2015, 27, 3424. 25914242 10.1002/adma.201500048

[52] M. Yang , Y. Zhou , Y. Zeng , C.‐S. Jiang , N. P. Padture , K. Zhu , Adv. Mater. (Deerfield Beach, Fla.) 2015, 27, 6363. 10.1002/adma.201502586 26414514

[53] X. Li , B. He , Z. Gong , J. Zhu , W. Zhang , H. Chen , Y. Duan , Q. Tang , Sol. RRL 2020, 4, 2000362.

[54] Y. Li , W. Xu , N. Mussakhanuly , Y. Cho , J. Bing , J. Zheng , S. Tang , Y. Liu , G. Shi , Z. Liu , Q. Zhang , J. R. Durrant , W. Ma , A. W. Y. Ho‐Baillie , S. Huang , Adv. Mater. 2022, 34, 2106280. 10.1002/adma.202106280 34741474

[55] C. Xu , X. Chen , S. Ma , M. Shi , S. Zhang , Z. Xiong , W. Fan , H. Si , H. Wu , Z. Zhang , Q. Liao , W. Yin , Z. Kang , Y. Zhang , Adv. Mater. 2022, 34, 2109998. 10.1002/adma.202109998 35112404

[56] L. Fan , Y. Ding , J. Luo , B. Shi , X. Yao , C. Wei , D. Zhang , G. Wang , Y. Sheng , Y. Chen , A. Hagfeldt , Y. Zhao , X. Zhang , J. Mater. Chem. A 2017, 5, 7423.

[57] H. Min , M. Kim , S.‐U. Lee , H. Kim , G. Kim , K. Choi , J. H. Lee , S. I. Seok , Science 2019, 366, 749. 31699938 10.1126/science.aay7044

[58] F. Fu , S. Pisoni , T. P. Weiss , T. Feurer , A. Wäckerlin , P. Fuchs , S. Nishiwaki , L. Zortea , A. N. Tiwari , S. Buecheler , Adv. Sci. 2018, 5, 1700675. 10.1002/advs.201700675 PMC5867048 29593970

[59] J. Barrier , R. E. Beal , A. Gold‐Parker , J. A. Vigil , E. Wolf , L. Waquier , N. J. Weadock , Z. Zhang , L. T. Schelhas , A. F. Nogueira , M. D. McGehee , M. F. Toney , Energy Environ. Sci. 2021, 14, 6394.

[60] C. G. Bischak , C. L. Hetherington , H. Wu , S. Aloni , D. F. Ogletree , D. T. Limmer , N. S. Ginsberg , Nano Lett. 2017, 17, 1028. 28134530 10.1021/acs.nanolett.6b04453

[61] J. Cho , P. V. Kamat , Chem. Mater. 2020, 32, 6206.

