# Supplementary material for: Tailoring Precursor‐Solvent Coordination Controls the Crystallization Kinetics and Nuclei Growth for Phase Homogenization in Wide‐Bandgap Perovskite Solar Cells
Source: Adv Sci (Weinh). 2025 Nov 14;12(48):e07660. doi: 10.1002/advs.202507660 (PMC12752567; doi:10.1002/advs.202507660)
Supplement: Supplementary file 1 — Supporting Information [file ADVS-12-e07660-s001.docx]

**Supplementary Information**

Tailoring Precursor-Solvent Coordination Controls the Crystallization Kinetics and Nuclei Growth for Phase Homogenization in Wide-Bandgap Perovskite Solar Cells

Saurabh Srivastava^#*1,7^, Sudhir Ranjan^#*2,7^, Harishankar Suman^3^, Shailesh Kumar Sah^4,7^, Shashikant Gupta^4^, Subhakar Mangam^5^, Shambhavi Rai^4^, Jayant Jain^5^, Srinivas Karthik Yadavalli^4,7^, Shivam Tripathi^1^, Anand Singh^4,6,7^, Raju Kumar Gupta^2,4,7,8^ and Ashish Garg^4,7*^

*^1^Department of Materials Science and Engineering, Indian Institute of Technology Kanpur, Kanpur 208016, India*

*^2^Department of Chemical Engineering, Indian Institute of Technology Kanpur, Kanpur 208016, India*

*^3^Department of Physics, Indian Institute of Technology Roorkee, Roorkee 247667, India*

*^4^Department of Sustainable Energy Engineering, Indian Institute of Technology Kanpur, Kanpur 208016, India*

*^5^Department of Materials Science and Engineering, Indian Institute of Technology Delhi, New Delhi 110016, India*

*^6^Department of Chemistry, Indian Institute of Technology Kanpur, Kanpur 208016, India*

*^7^Chandrakanta Kesavan Centre for Energy Policy and Climate Solutions, Indian Institute of Technology Kanpur, Kanpur 208016, India*

*^8^Centre for Environmental Science and Engineering, Indian Institute of Technology Kanpur, Kanpur 208016, India*

*^#^Both authors contributed equally to the manuscript*

*Corresponding author

*E-mail address:* Saurabh Srivastava (msaurabh@iitk.ac.in)

Sudhir Ranjan (sudran@iitk.ac.in)

Ashish Garg (ashishg@iitk.ac.in)

***This supplementary pdf file contains:***

Supplementary text

Figures S1 to S18

Tables S1 to S6

***Supplementary text:***

***Materials:***

FTO-coated glass substrates with a thickness of 500 nm and sheet-resistance of 7 Ω/□ were purchased from Global Nanotech Pvt. Ltd. SnO_2_ colloidal precursor (tin (IV) oxide, 15% in H_2_O colloidal dispersion) was purchased from Alfa Aesar. N, N-dimethylformamide (DMF), dimethyl sulfoxide (DMSO), chlorobenzene (CB), acetonitrile, ammonium chloride, Spiro-OMeTAD, 4-tert-butylpyridine (tBP) and bis (trifluoromethane) sulphonimide lithium salt (Li-TFSI) salt were purchased from Sigma-Aldrich. Perovskite precursor materials such as lead iodide (PbI_2_, 99% purity), lead bromide (PbBr_2_, 99% purity) and cesium iodide (CsI) were purchased from Tokyo Chemical Industry. Formamidinium iodide (FAI) was obtained from Greatcell Solar Materials. All the chemicals were used as received without any further modifications.

***Device fabrication:***

FTO-coated glass substrates of size 2.5 cm × 2.5 cm and FTO thickness of 500 nm were patterned by chemical etching with zinc dust and 2 M HCl solution. The patterned substrates were cleaned sequentially by ultrasonication in Hellmanex soap solution (2 vol%), deionized water, acetone and isopropanol, respectively, for 10 min each, followed by UV-Ozone treatment for 15 min. After cleaning, to deposit the SnO_2_ electron transport layer (ETL) on the substrates, SnO_2_ precursor solution was prepared by diluting the SnO_2_ colloidal solution with deionized water in 1:4 ratio, followed by ultrasonication for 30 min and then filtering it with a 0.22 µm PES filter. 40 nm thick SnO_2_ ETL was fabricated by spin coating the SnO_2_ precursor solution on the patterned substrates at 4000 rpm for 40 sec, rotated at a ramp rate of 2000 rpm s^-1^ and in 55-65% relative humidity (RH), followed by annealing at 170 °C for 30 min on a hot-plate. The wide-bandgap FACsPbIBr perovskite film was prepared as per a previous protocol [1]. The FA_0.8_Cs_0.2_Pb(I_0.7_Br_0.3_)_3_ (hereon used as I_7_Br_3_) precursor solution was prepared by dissolving 0.88 mmol FAI, 0.22 mmol CsI, 0.605 mmol PbI_2_ and 0.495 mmol PbBr_2_ in 1 mL mixed solvent of DMF and DMSO with a volume ratio of 4:1. The I_7_Br_3_ precursor solution with AC additive was prepared by dissolving desirable amount (in mol%) of AC in the above solution. All the perovskite precursor solutions were thoroughly mixed and stirred at 60 °C for 2 h before use. The perovskite thin films were spin-coated on SnO_2_ coated substrates via a two-step spin-coating process i.e. first at 1000 rpm for 10 sec and then at 5000 rpm for 30 sec, followed by annealing at 100 °C for 60 min on a hot plate for solvent evaporation and uniform crystallization of the perovskite films. The substrates were then cooled down to room temperature (RT). To fabricate hole transporting layer of Spiro-OMeTAD on the perovskite-coated substrates, a solution was prepared by dissolving 72.3 mg of Spiro-OMeTAD in 1 mL of chlorobenzene followed by stirring for 2 h at RT. Then 28.8 µL of 4-tert-butylpyridine solution and 17.7 µL of Li-TFSI solution (520 mg in 1 mL acetonitrile) were added to the HTL solution followed by stirring again for 30 min at 60 °C. The HTL was then spin-coated at 3000 rpm for 40 sec. The samples were then kept overnight in a dry box with 10% RH for overnight oxidation. Finally, the samples were transferred into a high-vacuum thermal evaporation chamber (10^-6^ mbar) for the deposition of ~100 nm Au electrodes using a shadow mask to achieve an active device area of 0.06 cm^2^.

***Thin film and device characterizations:***

For structural characterization, X-ray diffraction (XRD) patterns (θ-2θ scan) of the perovskite films coated substrates were obtained using monochromatic CuK_α_ radiation (λ = 1.5405 Å) in a PANanalytical Empyrean X-ray diffractometer. In-situ time-resolved temperature-dependent XRD was performed at a relative humidity of 20% in three different stages viz (I) at room temperature; (II) at room temperature under vacuum conditions; and (III) during thermal annealing under vacuum conditions. A vacuum of 10^-2^ millibar was applied during stages II and III. In stage I, measurement was done for 30 minutes at RT. In stages II and III, measurements were carried out for 20 and 90 minutes, respectively. Samples were heated from RT to 100°C at a ramp rate of 5°C/min and a holding time of 60 sec. Samples were scanned in the diffraction range of 5-16° at a scanning rate of ~ 3° /min and step size of 0.02°. The delay between two subsequent measurements was 30 sec.

The microstructural characterization was done using Nova NanoSEM 450 field-emission scanning electron microscope followed by determination of grain size distribution from FESEM images using ImageJ software. The dynamic light scattering (DLS) measurements were performed on Malvern Panalytical’s Zetasizer Nano ZS analyzer for dilute (0.1M) precursor solutions. The Steady-state photoluminescence and absorbance spectra of the perovskite films and PbI_2_ solution were collected using Jasco spectrophotometer at an excitation wavelength of 430 nm and UV-vis (Cary 5000) spectrophotometer. The time-resolved photoluminescence spectra were recorded using PicoQuant Fluotime 300 spectrophotometer. Surface topography measurements were recorded using Asylum MFP-3D atomic force microscope. The thickness of the perovskite films was determined using Bruker’s DektakXT stylus profilometer. The chemical states of the perovskite films were characterized by X-ray photoelectron spectroscopy (XPS) using PHI 5000 (Versa Probe II, FEI Inc.). For the elemental detection, the wavelength dispersive X-ray fluorescence (WD-XRF) measurements were done using Rigaku X-ray fluorescence spectrometer. The J-V characteristics of the as-fabricated perovskite photovoltaic devices were measured in the N_2_ glove box using a Keithley 2420 source meter and under a simulated AM 1.5G solar spectrum. Electrical impedance spectroscopy (EIS) measurements were performed using Autolab 302N, Metrohm potentiostat under dark conditions at DC voltage of 0.9 V and ac perturbation of 25 mV. NOVA 2.0 software was used to analyze the impedance spectra. Agilent 4294 A LCR meter was used to perform thermal admittance spectroscopy (TAS) of the devices. The temperature was controlled through a cryostat equipped with Lakeshore 3060 temperature controller. The elemental distribution of the films was determined using time-of-flight secondary ion mass spectrometry measurements on TOF-SIMS 5, IONTOF GmbH, Germany instrument. In-situ PL measurement was performed using a home-built setup inside the N_2_ glovebox at a photoexcitation wavelength of 470 nm using a laser. Photoexcitation was introduced through fiber optics while photoemission was collected and captured by the Ocean Optics USB2000 spectrophotometer.

***Computational details:***

We investigated the adsorption energies of DMSO and NH_4_ on the (0001) surface of hexagonal PbI_2_ (space group: P6_3_mc). The bulk lattice parameters of PbI_2_ unit cell were obtained from the Materials Project: $a=4.62 Å, b=4.62 Å, c=14.55 Å, \alpha=90^{\circ},\beta=90^{\circ}, and \gamma=120^{\circ}$. A (3 x 3 x 2) supercell containing four PbI_2_ layers along the (0001) directions was studied. To simulate the semi-infinite slab, the bottom layer was kept fixed, and a vacuum of 15 $Å$was added along the surface normal. First, we optimized the atomic structure of PbI_2_ slab, while the DMSO and the NH_4_ molecules were optimized in the gas phase. For DMSO, two adsorption configurations were considered: one via oxygen (O-binding) and the other via sulfur (S-binding). For NH_4_, only one stable configuration was identified. The relaxed adsorbed configurations of DMSO and NH_4_ on the PbI_2_ (0001) surface are shown in **Figure 1c-e** of the manuscript.

All density functional theory (DFT) calculations were performed using the Vienna ab initio simulation package (VASP).[1-4] Core–valence interactions were treated using the projector augmented wave (PAW) method, and the electron exchange–correlation energy was described using the generalized gradient approximation (GGA) with the Perdew–Burke–Ernzerhof (PBE) functional. [7] A plane-wave basis set with a kinetic energy cutoff of 520 eV was used to expand the electronic wave functions. Reciprocal space was sampled with a 2x2x1 k-point mesh. Gaussian smearing with a width of 0.05 was applied. The convergence criterion were set to ${10}^{-4}$eV for the total energy and 0.01 eV/$Å$ for the Hellmann–Feynman forces. The pseudopotentials included 14, 7, 6, 6, 4, 5, and 1 valence electrons for Pb, I, S, O, C, N, and H, respectively.

The adsorption energies (E_ad_) for DMSO and NH_4_ on PbI_2_ (0001) slab were calculated as follows:

$$E_{ad}=E\left( Slab+DMSO/NH_{4} \right) -E\left( Slab \right)-E(DMSO/NH_{4})$$


**Figure S1:** Chemical structure of AC.

**Figure S2: (a)** FTIR spectra showing PbI_2_ and AC additive interaction by stretching of the C=O peak. **(b)** FTIR spectra showing interactions of FAI and AC in DMF:DMSO **(c)** Photographs of vials containing FAI and FAI+AC solution in DMF and DMSO. **(d)** FTIR spectra showing FAI, AC and DMF:DMSO interactions by stretching of the C=N peak.

**Figure S3:** ^1^H NMR spectra of FAI, AC and FAI+AC mixture, highlighting the chemical shift variations that indicate the interaction between FAI and AC.

**Figure S4:** FTIR spectra showing PbBr_2_, AC and DMF: DMSO interaction.

**Table S1**: Peak position, Intensity and FWHM obtained from the XRD patterns of PbI_2_ and PbI_2_+AC.

| **Peaks** | **PbI_2_** | | | | **PbI_2_+AC** | | | | | |
| --- | --- | --- | --- | --- | --- | --- | --- | --- | --- | --- |
|  | **PbI_2_.DMSO** | | **(001)-PbI_2_** | | **PbI_2_.DMSO** | | **(001)-PbI_2_** | | **NH_4_Pb(I_x_Cl_1-x_)_3_** | |
|  | **2θ (°)/ Ints. (Counts)** | **FWHM (°)** | **2θ (°)/ Ints. (Counts)** | **FWHM (°)** | **2θ (°)/ Ints. (Counts)** | **FWHM (°)** | **2θ (°)/ Ints. (Counts)** | **FWHM (°)** | **2θ (°)/ Ints. (Counts)** | **FWHM (°)** |
| RT | 9.427/  466 | 1.2418 | -- | -- | 9.208/ 5306 | 0.1617 | 12.614/ 16935 | 0.1669 | 15.40/ 132 | 0.2582 |
| 70 °C  1 min | 9.492/ 213 | 0.9302 | 12.596/ 28588 | 0.2529 | 9.171/ 10596 | 0.1529 | 12.555/ 15109 | 0.1805 | 15.395/ 293 | 0.1963 |
| 70 °C  5 min | -- | -- | 12.621/ 42359 | 0.2419 | 9.227/ 11077 | 0.1516 | 12.617/ 23383 | 0.1841 | 15.388/ 411 | 0.2007 |
| 70 °C  10 min | -- | -- | 12.686/ 15822 | 0.2204 | -- | -- | 12.704/ 48481 | 0.2358 | -- | -- |

**Figure S5:** In-situ temperature-dependent XRD patterns of the control and target perovskite films at different time intervals.

**Figure S6:** XRD patterns of the control and target perovskite films at different time-interval of spin-coating.

**Figure S7:** Thermogravimetric analysis (TGA) curves of control and target precursors. Inset: Enlarged view of the 165-240 °C region highlighting the accelerated DMSO loss.

**Figure S8** Photographs of the control and target WBG perovskite precursors at t = 0, 5 and 30 minutes after anisole (antisolvent) dripping.

**Figure S9:** FWHM of the control and target perovskite films during the spin-coating and annealing steps as obtained from the fitting of the PL peaks.

**Figure S10:** Time-dependent in-situ PL spectra recorded during the spin-coating and annealing steps for the (a, b) control and (c, d) target perovskite films.

**Figure S11:** Schematic illustration of the sequential steps involved in the formation of wide-bandgap perovskite film with and without NH_4_Cl

**Step I:** Initial diffusion of halide (I^-^, Br^-^) ions leads to the formation of a PbX_2_ layer, which exhibits strong coordination with DMF and DMSO solvents.

**Steps II and III:** Rearrangement of the PbI_n_Br_1-n_ polyhedra occurs, resulting in the formation of 2H, 4H, 6H and 3C phases, accompanied by the gradual diffusion of bromide and iodide ions.

**Steps IV and V:** The presence of NH_4_Cl reduces the coordination of perovskite precursors with solvent, promoting the progressive development of corner-sharing PbI_n_Br_1-n_Cl_1-n_ polydedra. This ultimately facilitates halide homogenization and the formation of a uniform 3C phase.


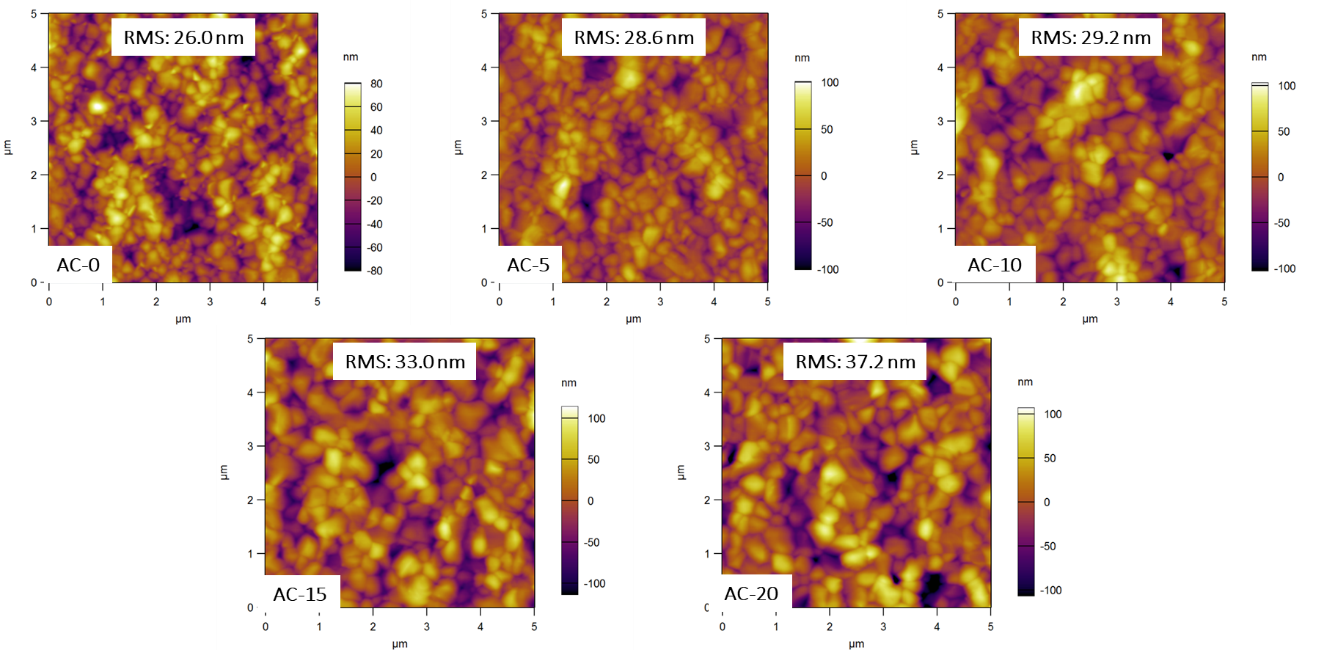


**Figure S12:** AFM images of the perovskite films with different concentrations of AC additive (AC-0, AC-5, AC-10, AC-15 and AC-20 samples).

**Figure S13:** FESEM images of the perovskite films with different concentrations of AC additive.

**Figure S14:** XRD patterns of the control and target perovskite films showing the shifting of (003) and (214) peaks after AC addition.

**Figure S15:** XRD patterns of the perovskite films with different concentrations (0, 5, 10, 15 and 20 mg per ml) of AC additive.

**Figure S16:** Absorbance and PL spectra of the control and target perovskite films.

**Figure S17:** Tauc plot depicting the bandgap of control and target samples.

**Figure S18:** PL spectra of the perovskite films with different concentrations of AC additive (AC-0, AC-5, AC-10, AC-15 and AC-20 samples).

**Table S2**: Decay fitting parameters determined from the TRPL spectra of the glass/perovskite films. TRPL curves were fitted using a tri-exponential decay equation of $I\left( t \right)= I_{0}+A_{1}\exp\left( \frac{-t}{\tau_{1}} \right)+A_{2}\exp\left( \frac{-t}{\tau_{2}} \right)+A_{3}exp(\frac{-t}{\tau_{3}})$.[2]

| **Perovskite** | **τ_1_ (ns)** | **A_1_ (kCnts/Chn)** | **τ_2_ (ns)** | **A_2_ (kCnts/Chn)** | **τ_3_ (ns)** | **A_3_ (kCnts/Chn)** | **τ_avg_ (ns)** |
| --- | --- | --- | --- | --- | --- | --- | --- |
| Control | 15.9 | 30.47 | 432.2 | 0.608 | 89.7 | 8.40 | 125.6 |
| Target | 21.8 | 2.97 | 740.0 | 0.133 | 150.7 | 1.164 | 297.5 |

**Figure S19:** XPS spectra of control and perovskite films showing the shift in **(a)** Pb peak **(b)** I peak and **(c)** Br peak.

**Figure S20:** C-AFM maps and corresponding line profile of control and target perovskite films.

**Figure S21: (a)** ToF-SIMS depth profiles showing halide ions distribution in as-deposited target and control perovskite films. **(b)** ToF-SIMS depth profile showing halide ions distribution in control perovskite before (dotted lines) and after (solid lines) annealing. **(c)** ToF-SIMS depth profile showing halide ions distribution in target perovskite before (dotted lines) and after (solid lines) annealing. **(d)** Ion-exchange mechanism between iodine and chlorine ions during nucleation stage in target perovskite films.

**Table S3**: Fitted parameters from the equivalent circuit of the Nyquist plots.

| **Sample** | **R_s_ (ohm cm^2^)** | **R_rec_ (ohm cm^2^)** |
| --- | --- | --- |
| Control | 0.094 | 4.854 |
| Target | 0.142 | 14.157 |

**Figure S22:** JV curve of the best-performing devices for different concentrations of AC additive (AC-0, AC-5, AC-10, AC-15 and AC-20 samples).

**Figure S23:** PV parameters of the devices with different concentrations of AC additive.

**Table S4**: PV Parameters of devices with different concentrations of AC additive.

| **AC additive concentration** | **V_oc_ (volts)** | **J_sc_ (mA/cm^2^)** | **FF (%)** | **PCE (%)** |
| --- | --- | --- | --- | --- |
| 0 | 1.134 | 18.69 | 71.43 | 15.14 |
| 5 | 1.188 | 19.52 | 69.42 | 16.10 |
| 10 | 1.224 | 19.83 | 73.93 | 17.96 |
| 15 | 1.195 | 19.61 | 70.89 | 16.62 |
| 20 | 1.185 | 19.19 | 69.14 | 15.73 |

**Figure S24:** EQE spectra (left axis) of the target device and the integrated J_sc_ values (right axis).

**Figure S25:** JV curves of the control and target devices in reverse (1.2 V -0.2 V) and forward (-0.2 V 1.2 V) scans.

**Table S5:** Chloride additive reported in the wide-bandgap perovskite literature.

| **Year** | **Perovskite** | **Device architecture** | **Additive** | **Solvent** | **V_oc_ (volts)** | **J_sc_ (mA/cm^2^)** | **FF (%)** | **PCE (%)** | **Ref.** |
| --- | --- | --- | --- | --- | --- | --- | --- | --- | --- |
| 2022 | Cs_0.17_FA_0.83_PbI_1.8_Br_1.2_ (E_g_: 1.74 eV) | FTO/ SnO_2_/ Perovskite/ Spiro-OMeTAD/ Ag | FACl | DMF:DMSO (intermediate polytypes) | 1.24 | 19.8 | 77.0 | 19.0 | [3] |
| 2023 | FA_0.83_Cs_0.17_Pb(I_0.6_Br_0.4_)_3_  (E_g_ ~ 1.80 eV) | ITO/ PTAA/ Al_2_O_3_/ Perovskite/ C_60_/ BCP/ Ag | MACl | DMF:DMSO (intermediate polytypes) | 1.25 | 17.2 | 79.0 | 17.0 | [4] |
| 2023 | FA_0.8_Cs_0.2_Pb(I_0.7_Br_0.3_)_3_  (E_g_ ~ 1.73 eV) | FTO/ SnO_2_/ Perovskite/ Spiro-OMeTAD/ Au | MACl | DMF:NMP (no intermediate polytypes) | 1.15 | 18.6 | 71.7 | 15.3 | [5] |
| **2024** | **FA_0.8_Cs_0.2_Pb(I_0.7_Br_0.3_)_3_**  **(E_g_ ~ 1.73 eV)** | **FTO/ SnO_2_/ Perovskite/ Spiro-OMeTAD/ Au** | **NH_4_Cl** | **DMF:DMSO (no 2H/4H intermediate polytypes)** | **1.22** | **19.8** | **73.9** | **17.9** | **This work** |

**Figure S26:** Change in the Urbach energy with time under light illumination for the control, control-T, AC-15 and AC-15-T perovskite films.

**Table S6**: Time-dependent Urbach energy (E_U_) values of perovskite films.

| **Time (hour)** | **Urbach energy (E_U_) (meV)** | |
| --- | --- | --- |
|  | **Control** | **Target** |
| 0 | 99 | 45 |
| 24 | 141 | 56 |
| 48 | 149 | 58 |
| 72 | 150 | 60 |
| 96 | 163 | 65 |

**Figure S27:** WD-XRF elemental spectra of the control and target perovskite films.

**Table S7**: Concentration and intensity of the elements detected through XRF measurements.

| **Perovskite** | **Elements** | | | | | | | |
| --- | --- | --- | --- | --- | --- | --- | --- | --- |
|  | **Pb** | | **I** | | **Br** | | **Cl** | |
|  | Conc. (mg/cm^2^) | Intensity (kcps) | Conc. (mg/cm^2^) | Intensity (kcps) | Conc. (mg/cm^2^) | Intensity (kcps) | Conc. (mg/cm^2^) | Intensity (kcps) |
| Control | 0.0684 | 3.301 | 0.286 | 1.514 | 0.0179 | 2.203 | 0 | 0 |
| Target | 0.0672 | 3.235 | 0.308 | 1.549 | 0.0227 | 2.793 | 0.0005 | 0.033 |

**References:**

1. Yu, Y., et al., *Synergistic effects of lead thiocyanate additive and solvent annealing on the performance of wide-bandgap perovskite solar cells.* ACS Energy Letters, 2017. **2**(5): p. 1177-1182.

2. Srivastava, S., et al., *Advanced spectroscopic techniques for characterizing defects in perovskite solar cells.* Communications Materials, 2023. **4**(1): p. 52.

3. Yu, F., et al., *Efficient and Stable Wide‐Bandgap Perovskite Solar Cells Derived from a Thermodynamic Phase‐Pure Intermediate.* Solar RRL, 2022. **6**(2): p. 2100906.

4. Shen, X., et al., *Chloride‐Based Additive Engineering For Efficient and Stable Wide‐Bandgap Perovskite Solar Cells.* Advanced Materials, 2023: p. 2211742.

5. Guaita, M.G., et al., *Influence of Methylammonium Chloride on Wide‐Bandgap Halide Perovskites Films for Solar Cells.* Advanced Functional Materials, 2023: p. 2307104.
